# Reverse Engineering of Modified Genes by Bayesian Network Analysis Defines Molecular Determinants Critical to the Development of Glioblastoma

**DOI:** 10.1371/journal.pone.0064140

**Published:** 2013-05-30

**Authors:** Brian W. Kunkle, Changwon Yoo, Deodutta Roy

**Affiliations:** 1 Department of Environmental and Occupational Health, Florida International University, Miami, Florida, United States of America; 2 Department of Biostatistics, Florida International University, Miami, Florida, United States of America; Texas Tech University, United States of America

## Abstract

In this study we have identified key genes that are critical in development of astrocytic tumors. Meta-analysis of microarray studies which compared normal tissue to astrocytoma revealed a set of 646 differentially expressed genes in the majority of astrocytoma. Reverse engineering of these 646 genes using Bayesian network analysis produced a gene network for each grade of astrocytoma (Grade I–IV), and ‘key genes’ within each grade were identified. Genes found to be most influential to development of the highest grade of astrocytoma, Glioblastoma multiforme were: COL4A1, EGFR, BTF3, MPP2, RAB31, CDK4, CD99, ANXA2, TOP2A, and SERBP1. All of these genes were up-regulated, except MPP2 (down regulated). These 10 genes were able to predict tumor status with 96–100% confidence when using logistic regression, cross validation, and the support vector machine analysis. Markov genes interact with NFkβ, ERK, MAPK, VEGF, growth hormone and collagen to produce a network whose top biological functions are cancer, neurological disease, and cellular movement. Three of the 10 genes - EGFR, COL4A1, and CDK4, in particular, seemed to be potential ‘hubs of activity’. Modified expression of these 10 Markov Blanket genes increases lifetime risk of developing glioblastoma compared to the normal population. The glioblastoma risk estimates were dramatically increased with joint effects of 4 or more than 4 Markov Blanket genes. Joint interaction effects of 4, 5, 6, 7, 8, 9 or 10 Markov Blanket genes produced 9, 13, 20.9, 26.7, 52.8, 53.2, 78.1 or 85.9%, respectively, increase in lifetime risk of developing glioblastoma compared to normal population. In summary, it appears that modified expression of several ‘key genes’ may be required for the development of glioblastoma. Further studies are needed to validate these ‘key genes’ as useful tools for early detection and novel therapeutic options for these tumors.

## Introduction

Astrocytomas are neoplasms of the brain that originate in a type of glial cell called an astrocyte. They are the most common glioma and their most aggressive form, glioblastoma multiforme, has a median survival of less than one year. While recent studies have characterized much of their basic biology, the major mechanisms behind the development of these tumors still remain unknown. Importantly, while some glioblastomas are thought to evolve from lower grade astrocytomas (secondary glioblastomas), most are thought to arise *de novo* (primary glioblastomas). There is a lack of clear understanding of the underlying molecular mechanisms of pathophysiology that drive the development of astrocytomas and this has hindered the progress of therapeutic development against it. Identifying molecular genetic differences between the typically benign lower grade astrocytomas (Grade I–II) malignant higher grade astrocytomas (Grade III–IV) could be an important step in better characterization of these highly malignant tumors. In addition, determination of the main pathways and genes involved in their development could provide for better therapies in the future.

Recent advances in high-throughput microarrays have produced a wealth of information concerning glioma biology. In particular, microarrays have been used to obtain differences in gene expression between normal non-tumor tissue and glioma tissue. Due to the relative rarity of gliomas, microarray data for these tumors is often the product of small studies, and thus pooling this data becomes desirable. Additionally, analysis of microarray data has been an evolving field as techniques such as cluster analysis, networking analysis and principal components analysis have been used in order to tease biologically relevant information from the large amount of data produced from microarrays. We chose to combine these analytic approaches through first combining available microarray data on gliomas using a meta-analysis approach, and then conducting Bayesian analysis on results of this meta-analysis. Our goal in this approach was to identify key genes and/or pathways that are critical in the development of astrocytic tumors. Through meta-analysis of 12 sub-studies which compared normal tissue to astrocytomas, we were able to identify a list of 554 genes which were differentially expressed in the majority of these studies. Many of the genes have in fact been implicated in development of astrocytoma, including EGFR, HIF-1α, c-Myc, WNT5A, and IDH3A. We then performed reverse engineering of our gene list using Bayesian network analysis. Four networks of genes were produced, one for each grade of Astrocytoma (Grade I–IV). Our results revealed the involvement of 8–18 key genes in the development and progression of astrocytoma depending on the grade of tumor. Alterations in the expression of eight to ten key genes may be required for the development of astrocytomas.

## Methods

Several steps were involved in our analysis, including: 1) identification of a significant set of over- and under-expressed genes through meta-analysis of several astrocytoma microarray studies; 2) enrichment analysis of the set of significant genes; 3) network analysis of the set of significant genes; and 4) investigation and validation of the network analysis. A more detailed description of these steps follows.

### Meta-analysis of Over- and Under-Expressed Genes In Astrocytoma Microarray Studies

Oncomine (Compendia Bioscience, Ann Arbor, MI), a web-based cancer microarray database, was used to perform meta-analysis of cancer vs. normal studies in Astrocytoma [1]. The goal of this analysis was to identify a set of significantly over-and under-expressed genes in Astrocytoma for further investigation. An Oncomine query for ‘Differential Analysis - Cancer vs. Normal Analysis’ and ‘Cancer Type - Brain and CNS Cancer’ was performed to identify studies that compared Astrocytoma to normal tissue. Pilocytic Astrocytoma (WHO Grade I), Diffuse Astrocytoma (WHO Grade II), Anaplastic Astrocytoma (WHO Grade III), and Primary and Secondary Glioblastoma Multiforme (WHO Grade IV) ‘sub-studies’ were chosen. Only studies analyzing microarray mRNA expression were used for the analysis. For purposes of this paper, ‘sub-studies’ are defined as studies on brain tumor sub-types within a larger overall study on brain tumors. Studies from our query that compared Astrocytic tumors to normal tissue were then selected for the meta-analysis. Oncomine ranks genes within each individual study based on a gene’s p-value compared to all other genes within the study. In meta-analysis, two heat-maps are returned: one for top over-expressed genes and one for top under-expressed genes. Genes in these heat-maps are ordered based on their median rank across the selected individual analyses. For our study, the top 600 significantly under-expressed and the top 600 significantly over-expressed genes from meta-analysis were narrowed to our ‘significant gene list’ by discarding all genes from these 1200 over- and under-expressed genes that were identified in 6 or less of the sub-studies. Thus, a gene was included in our final list of significant genes if it was identified as over- or under-expressed in at least 7 of the 10 sub-studies. This final set of genes was then subjected to enrichment and pathway analysis with several different tools.

### Gene Set Enrichment Analysis

FuncAssociate (Roth Laboratory, Harvard) and Ingenuity Pathway Analysis (IPA) (Redwood City, California) were used to identify pathways and other systems biology characteristics of our top set of genes. FuncAssociate is a web-based tool which performs a Fisher’s Exact Test to determine a list of Gene Ontology (GO) attributes that are over- (or under-) represented among a set of genes entered by the user [2]. GO Terms, curated by the Gene Ontology Consortium, identify significant cellular components (e.g. rough endoplasmic reticulum, ribosome), biological processes (e.g. signal transduction, pyrimidine metabolic process), and molecular functions (e.g. catalytic activity, binding, adenylate cyclase activity) of a set of genes [3]. Our significant gene list from Oncomine was entered into FuncAssociate for analysis. Settings were species: Homo sapiens; namespace: HGNC_Symbol; mode: ordered; simulations: 1000; over/under: both; and p-value cutoff: 0.05. The HGNC Symbol namespace setting resulted in our choosing the entire known human genome as our universe of comparison genes for the enrichment analyses.

IPA was also used to analyze our Oncomine gene list. This web-based program uses a manually curated database of findings from the scientific literature, along with data obtained from the Kyoto Encyclopedia of Genes and Genomes (KEGG), to analyze connections between genes, proteins, and other molecules. It also uses its own terminology for functional classifications of these molecules that is similar but not exact to the terminology used by GO. Enrichment analysis was performed using IPA’s “Core Analysis” function. Whereas GO Terms do not relate significant pathways of a set of genes, IPA Core Analysis does have this ability and therefore was used both to identify significant biological processes/molecular functions and to identify any pathways that were more commonly activated or inactivated in our set of genes. Significance of the identified processes and pathways is given by the right-tailed Fisher exact test p-value, meaning only overrepresented attributes are returned by IPA. The IPA default reference set of molecules, which includes all functionally-characterized molecules in IPA, was used as the universe of comparison genes. Several groups of processes are identified, including: biological functions (‘Bio Functions’), toxicological functions (‘Tox Functions’), and established pathways (‘Canonical Pathways’). The number of molecules from a set of data found to be in a pathway, divided by the total number of molecules in the identified canonical pathway is given.

### Reverse Engineering Bayesian Network Analysis of Differentially Expressed Genes in Astroctytic Tumors

Bayesian networks have been widely used and accepted in modeling molecular networks from microarray data [4], [5]. These networks are probabilistic graphical models that produce directed acyclic graphs (DAG) that represent a set of random variables and their conditional dependencies. Nodes of the DAG represent genes or other variables such as disease and are assumed to be conditionally independent of each other. The structures produced by Bayesian network analysis naturally represent causal hypotheses.

We used the software application Banjo (Duke University, NC) for probabilistic structure learning of static Bayesian networks from our steady state expression data from Oncomine [6]. Banjo performs structure inference using a local search strategy termed Bayesian Dirichlet equivalence (BDe) scoring metric for discrete variables. This strategy makes incremental changes in the structure aimed at improving the score of the structure. A score for the ‘best network’, influence scores for the edges of the best network, and a dot graphical layout file are returned as results of the search. The dot file is a DAG indicating regulation among genes and their possible influence on disease outcome.

The goal of this Bayesian analysis was to identify what may be the most critical genes for development of astrocytoma from our significant set of meta-analysis genes. This was accomplished by identifying a Markov blanket of each network output chosen as the ‘best network’ for each grade of astrocytoma. In a Bayesian network, the Markov blanket of any node *A* is its set of neighboring nodes composed of a nodes parents, children, and the parents of its children. This defined set of neighboring nodes shields node *A* from the rest of the network, and thus the Markov blanket of node *A* is the only knowledge needed to predict the behavior of node *A*.

Though its sensitivity is low, Banjo has been shown to have a very high positive predictive value for 100 plus case sets (regardless of the number of genes) composed of the type of ‘global’, steady-state gene data we analyzed [7]. For an overview of Bayesian network probability structures the reader is referred to Charniak 1991 [8]. Several other papers provide more detailed information on their construction and examples of their use with molecular modeling [5], [9]–[12].

To perform the analysis on our data, expression values for our significant set of genes were downloaded from Oncomine and loaded into Microsoft Excel. The top 100 over-expressed genes and top 100 under-expressed genes were then considered for analysis in Banjo. In order to increase our sample size, missing cases imputation was performed on cases with missing expression data for a particular gene using average of all expression values across the gene as the imputation. Cases without Grade identification and/or identified as non-tissue cases (i.e. cell lines) were excluded from the analysis. Studies from our meta-analysis with missing data for a large amount of genes were also excluded. The expression data for the remaining genes was then separated by Grade, discretized per study (due to Oncomine normalizing expression values per study), and combined for analysis in Banjo. Discretization of the data into three tiers of expression (under-, median-, and over-expressed) was performed using the programming software tool Perl. Assuming normally distributed data, the three tiers were selected based on a one standard deviation confidence interval (i.e. ∼68% of the values will have ‘median-expression’, with ∼16% of the values under-expressed and ∼16% of the values over-expressed). Discretized files were then run in Banjo for four separate analyses: 1) Normal Tissue vs. Grade I Pilocytic Astrocytoma cases, 2) Normal Tissue vs. Grade II Diffuse Astrocytoma cases, 3) Normal Tissue vs. Grade III Anaplastic Astrocytoma, and 4) Normal Tissue vs. Grade IV Glioblastoma Multiforme cases. Analyses was performed on the four Grades three separate times (three hours in length for each network search), with the ‘best network’ from these three runs being chosen as our ‘final best network’ for each Grade. Best network score significance was calculated using a log calculation of all three network scores, with a percent of the total score returned for each network.

### Predictive Analysis to Identify Key Markov Causal Genes of Each Grade of Astrocytoma

To assess the ability of Markov genes to distinguish between normal and tumor samples in our analysis, Genie, a software tool for analyzing Bayesian networks developed by the University of Pittsburgh [13], was used to predict the probability of developing Astrocytoma given certain expression states for its gene network. This predicts key Markov causal genes involved in the development of astrocytomas. In Bayesian network analysis this is done by learning the parameters of a given DAG structure. To accomplish this task, the discretized results files for each Grade of astrocytoma were loaded into the Genie software. Additionally, the Banjo network structure results were recreated in Genie. Genie’s ‘learn parameters’ function was then used to predict probabilities of outcomes for certain network structures. Given our small sample sizes, we did not allow a probability of 0 to be assigned to any result, choosing instead to use 0.01 for any probability calculated as 0. This allowed us to perform parameter assessment under the assumption that a low probability case may still have a very small chance of occurring in our data. Once our network parameters were established in Genie, we analyzed the probability of developing each grade of astrocytoma given differentially expressed states of the Markov Blanket genes of each grade using Bayes’ rule.

### Probability of Life Time Risk of Developing Astrocytoma Stage 4 - Glioblastoma from Joint Effects of Interactions between Markov Blanket Genes

To examine joint effects of interaction between Markov Blanket genes on the lifetime risk of developing Glioblastoma, we have used by Bayes’ theorem:
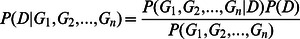
where *G*_1_, *G*_2_,…, *G*_n_ are expression level of selected Markov Blanket genes from the Bayesian network with the highest BDe score and D represent whether a subject have Glioblastoma or not. We used the 2005–2007 Surveillance, Epidemiology and End Results (SEER) calculated lifetime probability of diagnosis of cancer of the brain and other nervous system of 0.61% in normal population, i.e., P(*D*) = 0.0061 [14].

### Validation of Markov Key Causal Genes Predicted to be Involved in the Development of Astrocytoma by Statistical Methods

Several statistical methods were used to validate both the prediction capabilities and to assess the ability of our Markov genes to distinguish between normal and astrocytic tumor samples in our analysis. We performed prediction analysis by receiver operating characteristic (ROC) curve representing the Bayesian network discretized results; and validated our finding using linear regression, logistic regression, cross validation and support vector machine (SVM) analysis to assess the predictability of both the discretized and raw expression values of our Markov genes. Hierarchical Clustering was also performed on each set of Markov genes in order to further explore how these genes separated our set of non-tumor and tumor patients. These analyses were performed using both IBM SPSS Statistics 19.0 and Multi-Experiment Viewer (MeV) version 4.7.1.

### Literature Based Validation of Key Predicted Markov Causal Genes Involved in the Development of Glioblastoma Using Models Generated by Empirical Data

Several methods were used to investigate and validate both the prediction capabilities and the biological plausibility of our Markov network genes. They included literature and biological database searches, and curated gene and pathway analysis. The literature and database search of our Markov genes gathered information on gene cellular localization and function, and published research supporting the genes involvement in tumor formation by searching biological databases such The Human Gene Compendium’s Gene Cards (www.genecards.org), PubMed (www.pubmed.com), the Information Hyperlinked over Proteins (iHOP) Database (www.ihop-net.org), and the Glioblastoma Multiforme Database (GBMBase) (www.gbmbase.org).

In order to investigate existing literature and ontology based connections between our Markov gene lists we used programs in both IPA and PathJam [15]. The goal of these analyses was to investigate a) the quality of our network analysis findings in Banjo and Genie, and b) the biological relationships of our Markov genes from these analyses. The initial investigation was done using the Path Explorer feature of IPA. Path Explorer uses curated literature and experimental evidence of biochemical interactions to produce networks of existing connections between a set of user imputed genes. This function was used to search for any existing connections among the Grade IV Astrocytoma Markov Key Causal Genes.

IPA’s Core Analysis was then performed on these same Grade IV Astrocytoma Markov Genes in order to produce connections for a set of genes independent of their established pathways. This analysis generated gene networks by including genes in pathways of the inputted gene list. Networks are ordered in importance by an IPA-defined significance score. Settings for this analysis were Direct and Indirect Relationships, All Data Sources, All Species, and All Tissues & Cell Lines. The Human Genome U133 Plus 2.0 Array (19,079 genes) was selected as our reference universe of genes as it contained the largest gene set from our meta-analysis and was used in 2 of the 5 meta-analysis studies used for our Banjo analysis. The top identified network from the Core Analysis was compared to our Banjo/Genie generated results. Complementary to this Core Analysis’s production of top biological and disease related functions was our investigation of our Markov genes using PathJam [15]. This public server-based tool allows for interpretation of gene lists by integrating pathway-related annotations from several public sources including Reactome, KEGG, NCBI Pathway Interaction Database, and Biocarta. Using this tool we were able to produce interactive graphs linking all four Astrocytoma Markov gene lists with pathway annotations, allowing for graphical pathway investigation into our gene lists.

## Results

### Meta-analysis of Differentially Expressed Genes in Astrocytic Tumors

A total of 12 studies (with 27 sub-studies) conducting cancer vs. normal analysis on ‘Brain and CNS Cancer’ were identified in Oncomine. Non-astrocytic tumor studies and studies analyzing DNA (i.e. acCGH arrays) were then discarded, leaving seven studies (10 sub-studies) on astrocytoma for the meta-analysis. These 10 sub-studies are listed in Table 1.

**Table 1:** List of Oncomine studies in meta-analysis of Astrocytoma vs. Normal Studies.

Oncomine Study ID, *Publication Journal*, Date	Study Astrocytoma Type*	n (tumor/normal)
**Bredel Brain 2, ** ***Cancer Res*** **, 2005 ** [**16**]	Glioblastoma	27/4
**Gutmann Brain, ** ***Cancer Res*** **, 2002 ** [**17**]	Pilocytic Astrocytoma	8/3
**Lee Brain, ** ***Cancer Cell*** **, 2006 ** [**18**]	Glioblastoma	22/3
**Liang Brain, ** ***Proc Natl Acad Sci USA*** **, 2005 ** [**19**]	Glioblastoma	30/3
**Rickman Brain, ** ***Cancer Res*** **, 2001 ** [**20**]	Astrocytoma	45/6
**Shai Brain, ** ***Oncogene*** **, 2003 ** [**21**]	Astrocytoma	5/7
**Shai Brain, ** ***Oncogene*** **, 2003 ** [**21**]	Glioblastoma	27/7
**Sun Brain, ** ***Cancer Cell*** **, 2006 ** [**22**]	Anaplastic Astrocytoma	19/23
**Sun Brain, ** ***Cancer Cell*** **, 2006 ** [**22**]	Diffuse Astrocytoma	7/23
**Sun Brain, ** ***Cancer Cell*** **, 2006 ** [**22**]	Glioblastoma	81/23

* All studies are Astrocytoma tissue type vs. normal tissue.

The top 600 significantly over-expressed and top 600 significantly under-expressed genes were identified from a total of 10 ‘sub-studies’. The narrowing of the initial list of 1200 genes produced a total of 646 genes for further analysis (372 significantly over-expressed genes and 274 significantly under-expressed genes). A list of these genes can be found in Table S1 and Table S2 (See File S1). It should be noted that Primary and Secondary Glioblastomas were separated within only one of the nine studies identified as Astrocytoma in Oncomine (Bredel: 27 Primary vs. 2 Secondary Glioblastomas). Therefore, separation of these subtypes of Glioblastomas was not considered in our study.

### Gene Set Enrichment Analysis of Differentially Expressed Genes in Astrocytic Tumors

In order to identify significant biological processes, molecular functions, and pathways of the final set of 646 genes, we conducted enrichment analysis on this set of genes. As described in the methods, two separate programs were used for this analysis: FuncAssociate and IPA.

### FuncAssociate Results

FuncAssociate identified 60 GO Terms as being over-represented and 1 GO Term as being under-represented among our set of 314 over-expressed genes (see Table S1 and Table S2 in File S1). Several significant processes were related to nervous system processes (axon part, postsynaptic density, synapse part, synaptic transmission, neuron projection), developmental processes (cell part morphogenesis, cellular component morphogenesis, regulation of anatomical structure morphogenesis, anatomical structure morphogenesis, regulation of developmental process, anatomical structure development, development process), and several cellular processes associated with cancer (cell adhesion, biological adhesion, regulation of cell proliferation, regulation of apoptosis) (see Table S3 in File S1). Several genes involved in developmental processes have been linked to brain tumor development. A total of 147 genes out of 646 differentially expressed genes in astrocytic tumors were categorized in the GO developmental process terms listed above. Several of these genes, including MYC, EGFR, HIF1A, HGF, APOE, TIMP3, and WNT5A have been identified as being important to development of astrocytoma.

### Ingenuity Pathway Analysis Results

**Figure 1:**
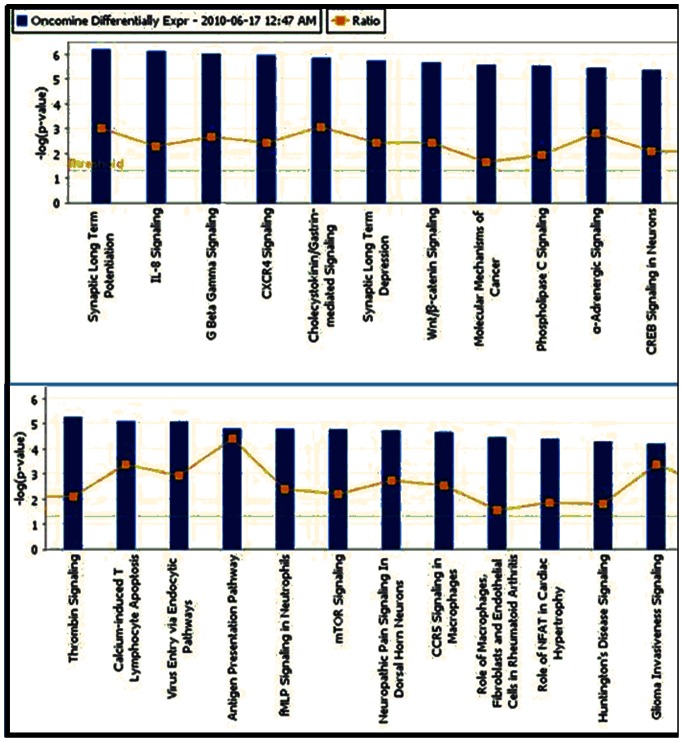
Top Canonical Pathways for Astrocytoma differentially expressed genes. The threshold line denotes the cutoff for significance (p-value = 0.05). Ratio is the number of molecules in the input list vs. the total number of molecules in the function.

IPA produced similar and contrasting results to the above analysis using FuncAssociate. Top Canonical Pathways identified for the over-expressed gene list include: ‘Synaptic Long Term Potentiation’ (p-value: 6.25E-07; Ratio of molecules in pathway from user list/total molecules in pathway: 16/113), ‘IL-8 Signaling’ (p-value: 7.41E-07; Ratio: 20/186), ‘G Beta Gamma Signaling’ (p-value: 9.48E-07; Ratio: 15/119), ‘CXCR4 Signaling’ (p-value: 1.1E-06; Ratio: 19/167), and ‘Cholecystokinin/Gastrin-mediated Signaling’ (p-value: 1.39E-06; Ratio: 15/104) (Figure 1). Several pathways known to be important to glioma development were also at the top of the significant canonical pathways list, including ‘WNT/beta-Catenin Signaling’ (CD44, CDH2, DVL3, LRP1, MYC, SOX4, SOX9, SOX13, TCF3, TCF4, TLE3, WNT5A) and ‘mTOR Signaling’ (EIF3B, EIF3E, EIF3F, EIF4A1, HIF1A, PRKD1, RHOC, RND2, RND3). Confirming our gene list as involved with brain tumor development, ‘Glioma Invasiveness Signaling’ (CD44, F2R, ITGAV, MMP9, RHOC, RND2, RND3, TIMP3, TIMP4) and ‘Glioblastoma Multiforme Signaling’ (CDK6, CDKN1A, EGFR, ITPR2, MYC, RHOC, RND2, RND3, TCF3, WNT5A) were returned as significant pathways as well.

**Figure 2:**
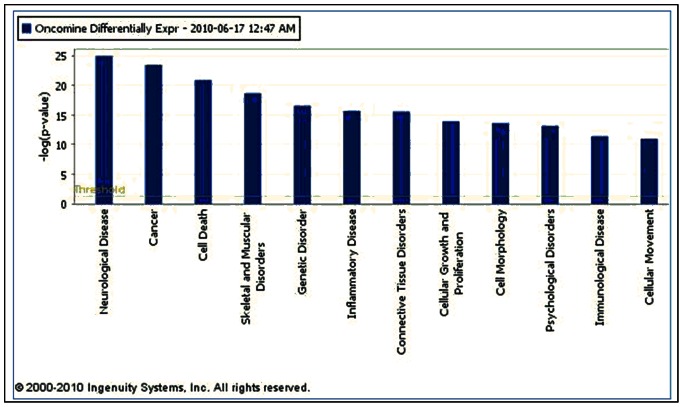
Top Biological Functions for Astrocytoma differentially expressed genes. The threshold line denotes the cutoff for significance (p-value = 0.05).

IPA Core Analysis also returns what are termed ‘Top Bio Functions’, grouped into three categories: Diseases and Disorders, Molecular and Cellular Functions, and Physiological System Development and Function. Significant functions are returned with their associated p-value and # of input molecules in the function. The top 5 Disease and Disorders for our list of 554 astrocytoma differentially expressed genes were: ‘Neurological Disease’ (p-value: 1.17E-25–4.98E-04; 270 molecules from our list), ‘Cancer’ (3.83E-24–5.61E-04; 240 molecules), ‘Skeletal and Muscular Disorders’ (2.32E-19–4.42E-04; 206 molecules), ‘Genetic Disorder’ (3.04E-17–5.27E-04; 354 molecules), and ‘Inflammatory Disease’ (2.20E-16–4.92E-04; 195 molecules) (Figure 2). As shown in the Figure 2, the top 5 Molecular and Cellular Functions were ‘Cell Death’ (1.36E-21–5.81E-04; 205 molecules), ‘Cellular Growth and Proliferation’ (1.23E-14–4.48E-04; 203 molecules), ‘Cell Morphology’ (2.51E-14–4.82E-04; 99 molecules), ‘Cellular Movement’ (1.08E-11–4.58E-04; 116 molecules), and ‘Cell Cycle’ (5.83E-11–5.61E-04; 91 molecules).

The top 5 Physiological System Development and Functions were ‘Tissue Development’ (1.65E-09–5.79E-04; 105 molecules), ‘Skeletal and Muscular System Development and Function’ (1.67E-09–2.96E-04; 54 molecules), ‘Tissue Morphology’ (7.04E-08–1.35E-04; 78 molecules), ‘Nervous System Development and Function’ (1.48E-07E –3.65E-04; 96 molecules), and ‘Behavior’ (1.67E-07–3.09E-04; 47 molecules). Figure 1 shows these top Bio Functions in order of significance. When interpreting these results, it is important to keep in mind that the p-values refer to the High Level Functions rather than to individual Lower-Level Functions, and therefore, if a High Level Function contains two or more specific Lower-Level Functions, a range of significances is displayed.

**Figure 3:**
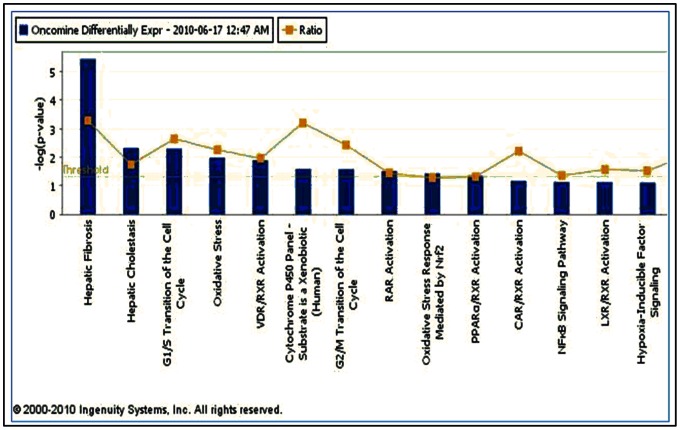
Top Toxicological Functions for Astrocytoma differentially expressed genes. The threshold line denotes the cutoff for significance (p-value = 0.05). Ratio is the number of molecules in the input list vs. the total number of molecules in the function.

Core Analysis also produces Top Toxicity Profiles. The Top 5 profiles for our 554 differentially expressed genes were ‘Hepatic Fibrosis’ (p-value: 3.59E-06; Ratio of molecules: 13/85), ‘Hepatic Cholestasis’ (p-value: 4.77E-03; Ratio: 11/135), ‘G1/S Transition of the Cell Cycle’ (p-value: 5.02E-03; Ratio: 6/49), ‘Oxidative Stress’ (p-value: 1.05E-02; Ratio: 6/57), and ‘VDR/RXR Activation’ (p-value: 1.28E-02; Ratio: 7/77) (Figure 3).

### Reverse Engineering Bayesian Network Analysis of Differentially Expressed Genes in Astrocytic Tumors

Four separate analyses were run in Banjo in order to search for genes critical for Grade I, II, III and IV Astrocytoma development. As discussed in the methods, studies and/or genes with missing expression data were excluded from the network analysis. Studies removed for both analyses were Bredel 2005, Liang 2005, and Rickman 2001. Additionally, Gutmann 2002 was removed from the Grade IV analyses as it did not contain Grade IV tumors. Genes were removed from our top 200 genes list (100 over- and 100 under-expressed genes) for each analyses based on availability per Grade. A total of 77 genes were removed for Grade 1, 68 for Grade 2, and 23 each for Grades 3 and 4.

**Figure 4:**
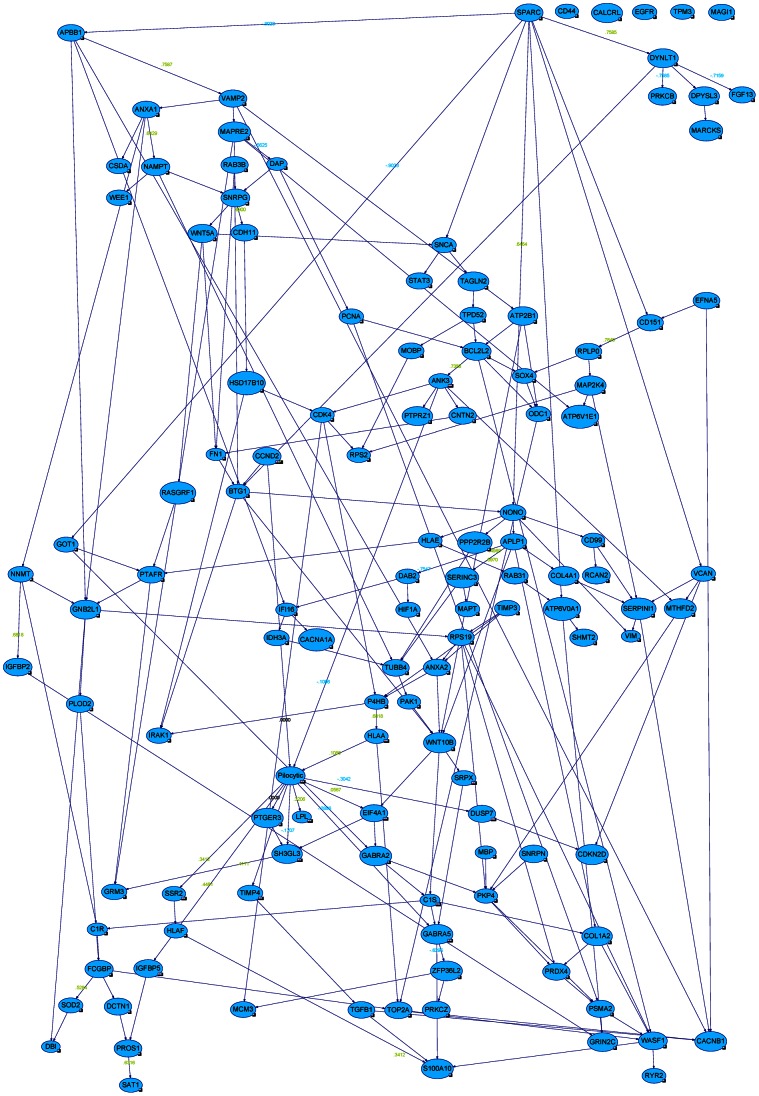
Bayesian networks with probabilistic structure learning from changes in the expression of modified genes in pilocytic astrocytic tumors. We used a program called Bayesian Network Inference with Java Objects (Banjo) to analyze the modified genes in pilocytic tumors. We ran our data through Banjo a total of 3 different machines. Each machine ran Banjo for three hours. The ‘best network’, with the highest BDE score that predicted the genes involved in stage 1 of astrocytoma (pilocytic tumor) is shown.

**Figure 5:**
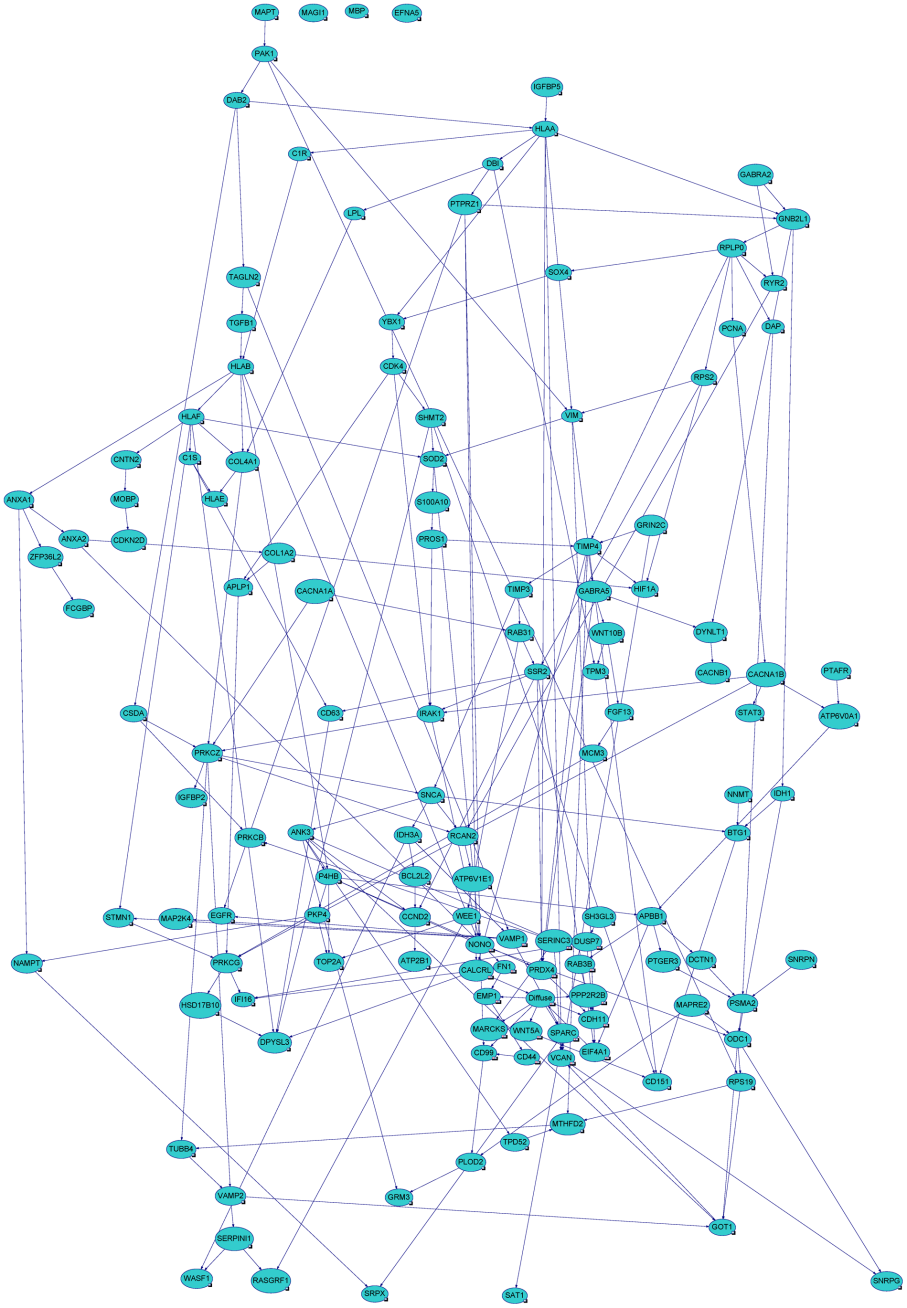
Bayesian networks with probabilistic structure learning from changes in the expression of modified genes in diffuse astrocytic tumors. The ‘best network’, with the highest BDE score using a Bayesian Network Inference with Java Objects (Banjo) program predicting the genes involved in stage 2 of astrocytoma (diffuse tumor) is shown.

**Figure 6:**
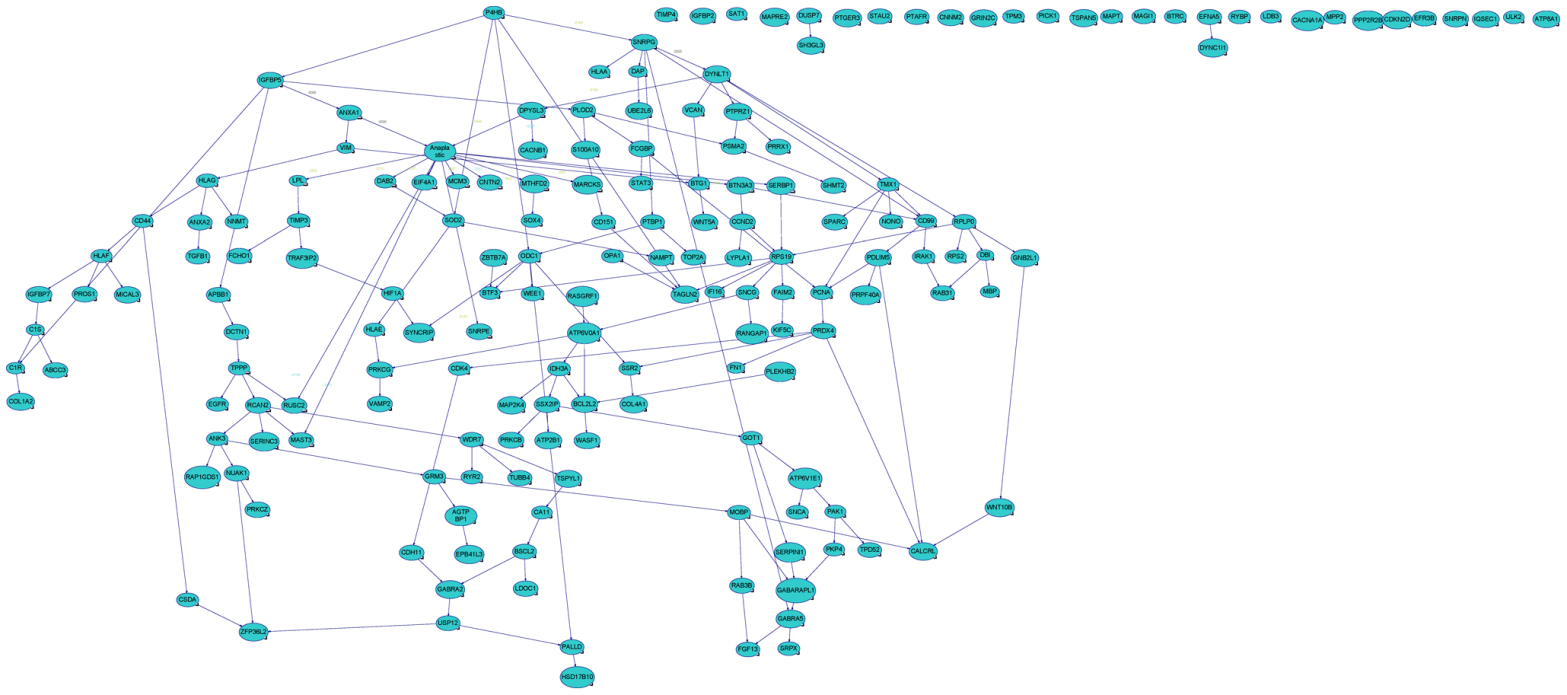
Bayesian networks with probabilistic structure learning from changes in the expression of modified genes in anaplastic astrocytic tumors. The ‘best network’, with the highest BDE score using a Bayesian Network Inference with Java Objects (Banjo) program predicting the genes involved in stage 3 of astrocytoma (anaplastic tumor) is shown.

**Figure 7:**
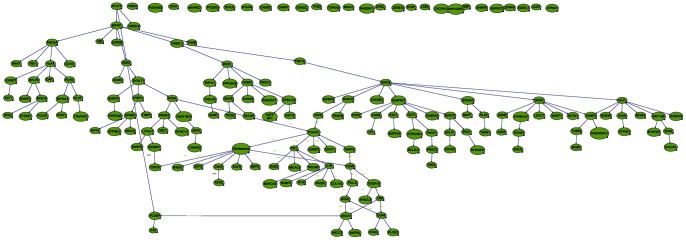
Bayesian networks with probabilistic structure learning from changes in the expression of modified genes in glioblastoma. The ‘best network’, with the highest BDE score using a Bayesian Network Inference with Java Objects (Banjo) program predicting the genes involved in stage 4 of astrocytoma (glioblastoma) is shown.

**Figure 8:**
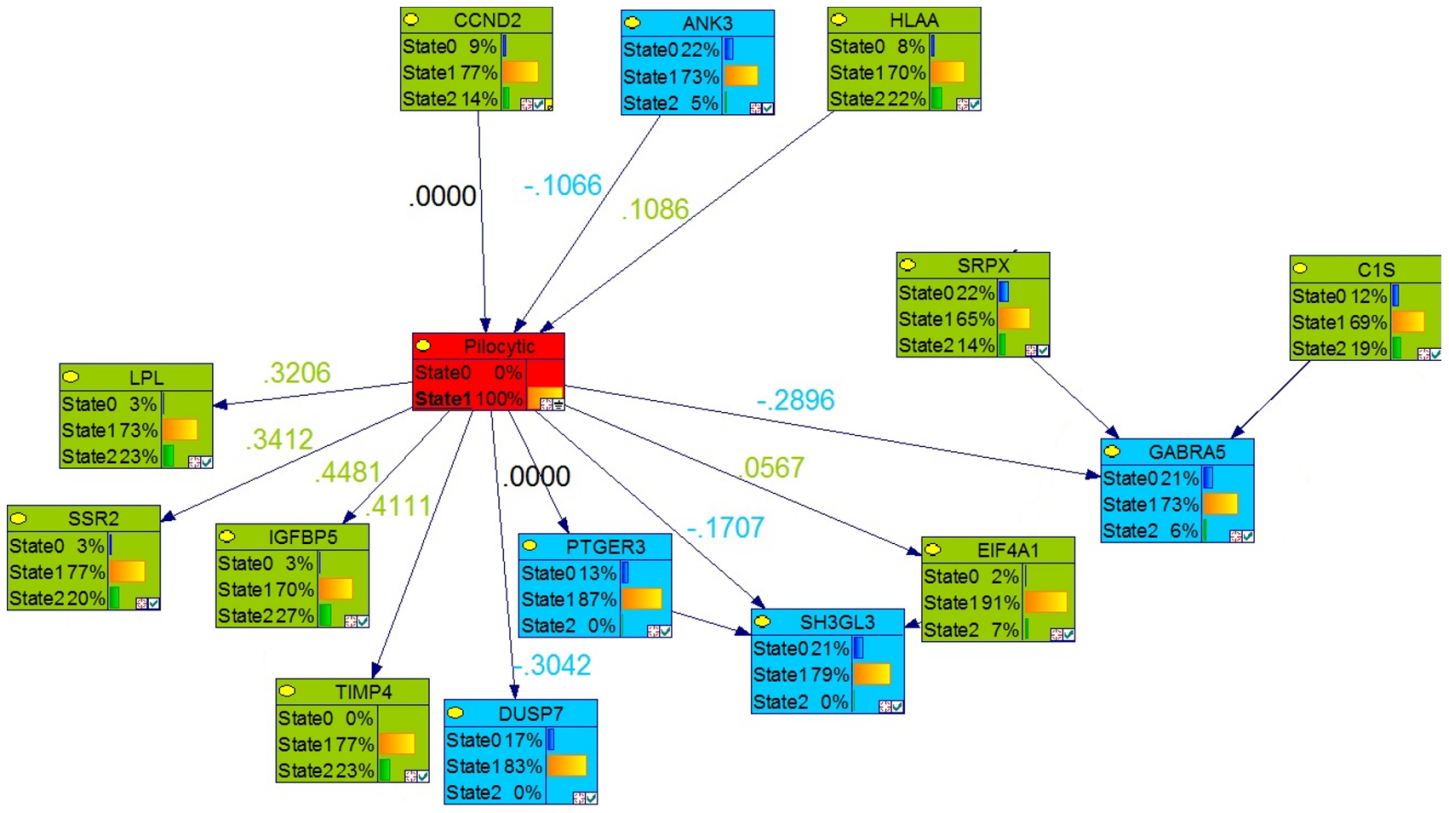
Markov blanket genes for Bayesian network of Pilocytic Astrocytoma. Green shade genes are overexpressed genes and blue shade genes are under expressed genes from the Oncomine meta-analysis.

We used Banjo for probabilistic structure learning of static Bayesian networks from our steady state expression data from these modified genes in each grade. Banjo was allowed to perform the structure inference analysis for 3 independent structure searches and each search was run for 3 hours for each grade. These three independent Banjo structure produced a network with BDe score (see a representative Figures 4–8 and Table 2). Please see Table 2 for sample size, sample statistics and significance of search score results of Bayesian network analysis. BDe score helped us to identify the ‘best network’, that predicted the genes involved in each stage of astrocytoma and the network with the highest BDE score was selected for further Markov Blanket analysis. Most of the modified genes were in the network, except CD44, CALCRL, EGFR, TPM3, and MAGI1 in pilocytic (Figure 4); MAGI1, MBP, and EFNA5 in diffuse (Figure 5); DYNLT1, TIMP4, IGFBP2, SAT1, MAPRE2, SH3GL3, PTGER3, STAU2, PTAFR, CNNM2, DUSP7, GRIN2C, TPM3, PICK1, TSPAN5, MAPT, MAGI1, BTRC, DYNC1I1, RYBP, LDB3, CACNA1A, MPP2, PPP2R2B, CDKN2D, EFR3B, SNRPN, EFNA5, IQSEC1, ULK2, and ATP8A1 in anaplastic (Figure 6); and PLEKHB2, OPA1, MAPRE2, PTGER3, STAU2, PTAFR, CNNM2, DUSP7, GRIN2C, TPM3, TSPAN5, MAGI1, RASGRF1, BTRC, ZBTB7A, RYBP, LDB3, CACNA1A, RAP1GDS1, MBP, SNRPN, SERINC3, EFNA5, IQSEC1, ULK2, and ATP8A1 in glioblastoma tumors (Figure 7).

**Table 2:** Sample statistics and significance of search score results of Bayesian network analysis.

	Studies in Analysis	Normal	Tumor	Genes Analyzed for Network	Bayesian Analysis Network Score Significance*		
					Search 1	Search 2	Search 3
**Normal vs. Grade 1**	Bredel, Gutmann, Rickman	13	30	122	1.37%	98.16%	0.46%
**Normal vs. Grade II**	Rickman, Shai, Sun	36	14	131	9.11%	6.62%	84.62%
**Normal vs. Grade III**	Bredel, Shai, Sun	34	23	176	1.98%	0.04%	99.95%
**Normal vs. Grade IV**	Bredel, Shai, Sun	34	137	176	0.00%	0.00%	99.99%

* Significance score for each network equals percent of total score for all three networks combined.

### Identification of Key Genes Involved in Each Stage of Astrocytoma by Markov Blanket Genes

**Figure 9:**
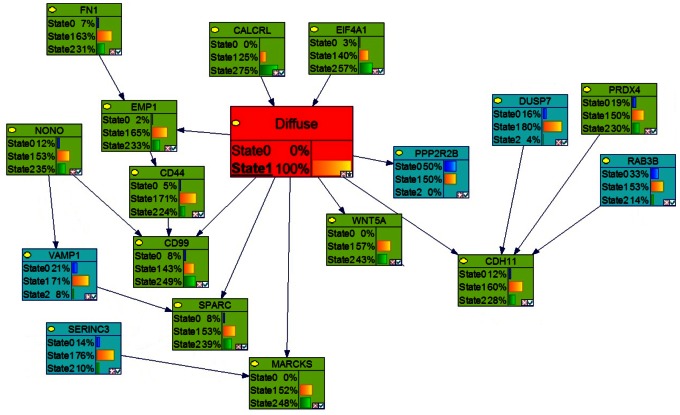
Markov blanket genes for Bayesian network of Diffuse Astrocytoma. Green shade genes are overexpressed genes and blue shade genes are under expressed genes from the Oncomine meta-analysis.

**Figure 10:**
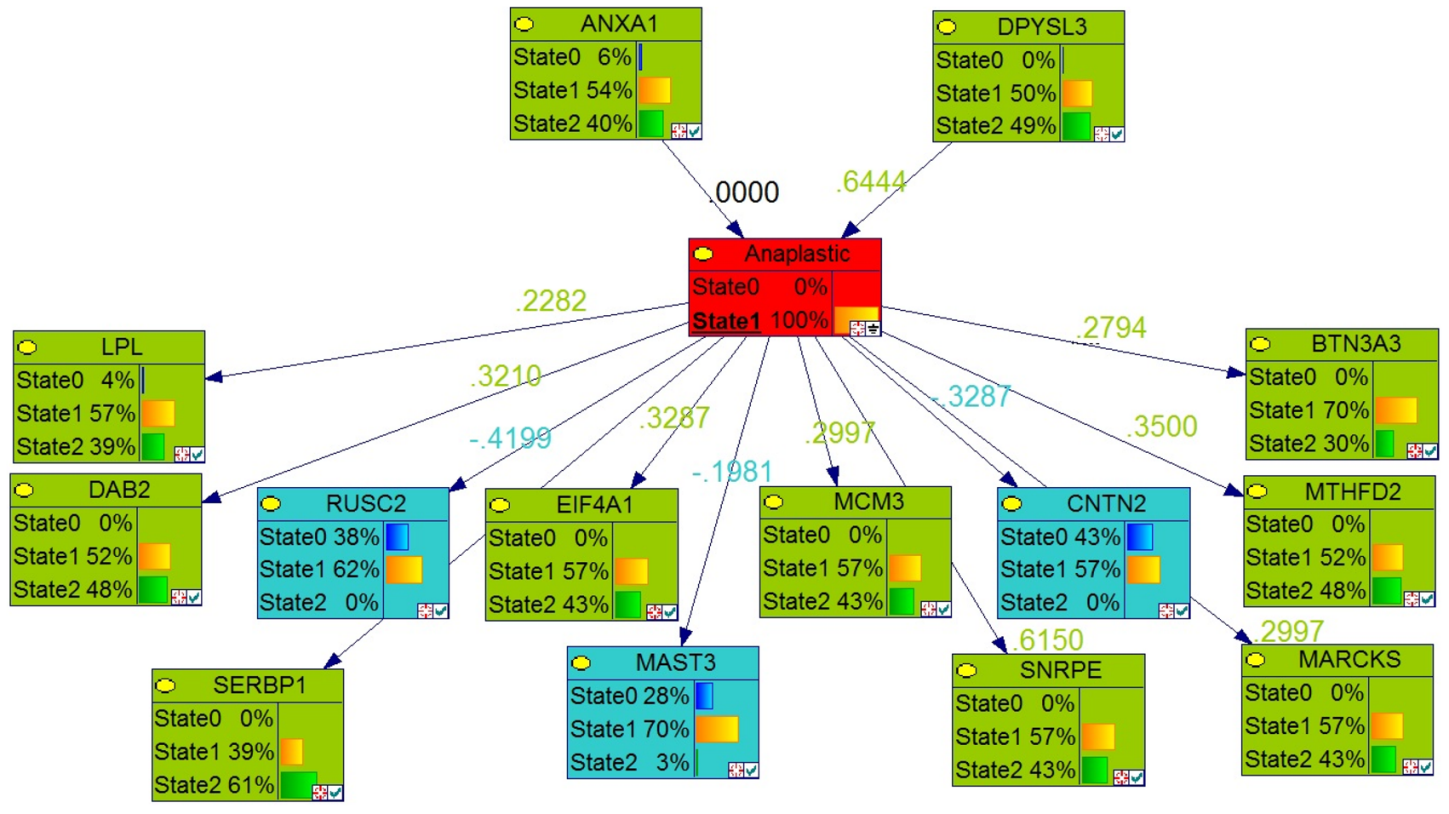
Markov blanket genes for Bayesian network of Anaplastic Astrocytoma. Green shade genes are overexpressed genes and blue shade genes are under expressed genes from the Oncomine meta-analysis.

**Figure 11:**
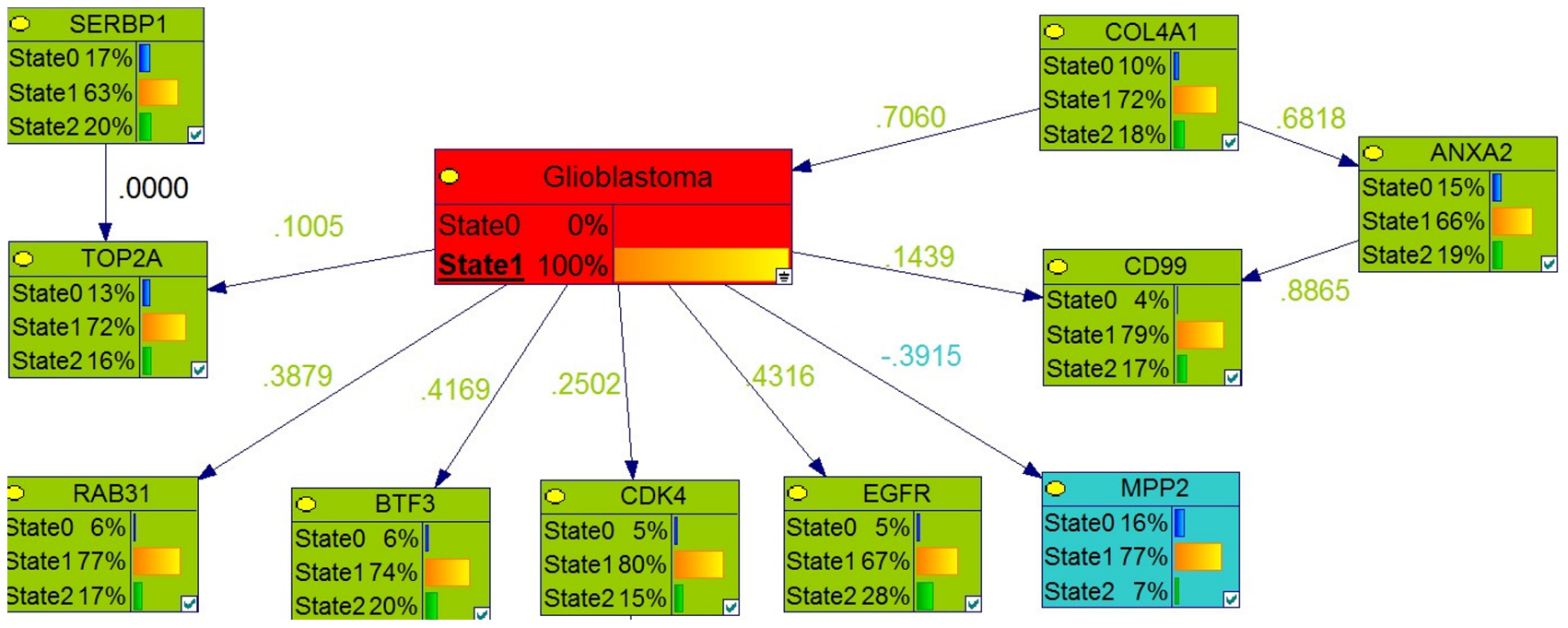
Markov blanket genes for Bayesian network of Glioblastoma Multiforme. Green shade genes are overexpressed genes and blue shade genes are under expressed genes from the Oncomine meta-analysis.

We used the Bayesian network genes of each stage of astrocytoma to further identify the most critical genes involved in the development of astrocytoma. This was accomplished by identifying a set of Markov Blanket genes from each gene network. This allowed us to define a set of neighboring genes that are sufficient to predict the probability of developing astrocytoma (Figures 8–11) and is summarized in Table 3. DAG structures for Markov genes of each grade of Astrocytoma are shown in Figures 8–11. Grade I Pilocytic Astrocytoma’s Markov blanket genes were: IGFB5, TIMP4, SSR2, LPL, DUSP7, GABRA5, SH3GL3, C1S, WNT10B, SRPX, ANK3, HLAA, EIF4A1, PTGER3, and CCND2 (Figure 8). Grade II Diffuse Astrocytoma’s Markov blanket genes were: FN1, MARCKS, PRDX4, NONO, SPARC, WNT5A, CD44, EIF4A1, CD99, CALCRL, EMP1, VCAN, CDH11, VAMP1, RAB3B, DUSP7, PPP2R2B, and SERINC3 (Figure 9). Grade III Anaplastic Astrocytoma’s Markov blanket genes were: LPL, MARCKS, SERBP1, DPYSL3, SNRPE, EIF4A1, ANXA1, MCM3, BTN3A3, MTHFD2, DAB2, RCAN2, RUSC2, TPPP, MAST3, and CNTN2 (Figure 10). Grade IV Glioblastoma Multiforme’s Markov blanket genes were: COL4A1, EGFR, BTF3, MPP2, RAB31, CDK4, CD99, ANXA2, TOP2A, and SERBP1 (Figure 11).

**Table 3:** Comparison of Markov Blanket genes across Grades I to IV Astrocytoma.

PILOCYTIC	DIFFUSE	ANAPLASTIC	GLIOBLASTOMA
*ANK3*	*ANK3*	*ANK3*	*ANK3*
**ANXA1**	**ANXA1**	**ANXA1**	**ANXA1**
**ANXA2**	**ANXA2**	**ANXA2**	**ANXA2**
**BTF3**	**BTF3**	**BTF3**	**BTF3**
**BTN3A3**	**BTN3A3**	**BTN3A3**	**BTN3A3**
**C1S**	**C1S**	**C1S**	**C1S**
**CALCRL**	**CALCRL**	**CALCRL**	**CALCRL**
**CCND2**	**CCND2**	**CCND2**	**CCND2**
**CD44**	**CD44**	**CD44**	**CD44**
**CD99**	**CD99**	**CD99**	**CD99**
**CDH11**	**CDH11**	**CDH11**	**CDH11**
**CDK4**	**CDK4**	**CDK4**	**CDK4**
*CNTN2*	*CNTN2*	*CNTN2*	*CNTN2*
**COL4A1**	**COL4A1**	**COL4A1**	**COL4A1**
*DAB2*	*DAB2*	*DAB2*	*DAB2*
*DPYSL3*	*DPYSL3*	*DPYSL3*	*DPYSL3*
*DUSP7*	*DUSP7*	*DUSP7*	*DUSP7*
**EGFR**	**EGFR**	**EGFR**	**EGFR**
**EIF4A1**	**EIF4A1**	**EIF4A1**	**EIF4A1**
**EMP1**	**EMP1**	**EMP1**	**EMP1**
**FN1**	**FN1**	**FN1**	**FN1**
*GABRA5*	*GABRA5*	*GABRA5*	*GABRA5*
**HLAA**	**HLAA**	**HLAA**	**HLAA**
**IGFBP5**	**IGFBP5**	**IGFBP5**	**IGFBP5**
**LPL**	**LPL**	**LPL**	**LPL**
**MARCKS**	**MARCKS**	**MARCKS**	**MARCKS**
*MAST3*	*MAST3*	*MAST3*	*MAST3*
**MCM3**	**MCM3**	**MCM3**	**MCM3**
*MPP2*	*MPP2*	*MPP2*	*MPP2*
**MTHFD2**	**MTHFD2**	**MTHFD2**	**MTHFD2**
**NONO**	**NONO**	**NONO**	**NONO**
*PPP2R2B*	*PPP2R2B*	*PPP2R2B*	*PPP2R2B*
**PRDX4**	**PRDX4**	**PRDX4**	**PRDX4**
**PTGER3**	**PTGER3**	**PTGER3**	**PTGER3**
**RAB31**	**RAB31**	**RAB31**	**RAB31**
*RAB3B*	*RAB3B*	*RAB3B*	*RAB3B*
*RCAN2*	*RCAN2*	*RCAN2*	*RCAN2*
*RUSC2*	*RUSC2*	*RUSC2*	*RUSC2*
**SERBP1**	**SERBP1**	**SERBP1**	**SERBP1**
*SERINC3*	*SERINC3*	*SERINC3*	*SERINC3*
*SH3GL3*	*SH3GL3*	*SH3GL3*	*SH3GL3*
**SNRPE**	**SNRPE**	**SNRPE**	**SNRPE**
**SPARC**	**SPARC**	**SPARC**	**SPARC**
**SRPX**	**SRPX**	**SRPX**	**SRPX**
**SSR2**	**SSR2**	**SSR2**	**SSR2**
**TIMP4**	**TIMP4**	**TIMP4**	**TIMP4**
**TOP2A**	**TOP2A**	**TOP2A**	**TOP2A**
*TPPP*	*TPPP*	*TPPP*	*TPPP*
*VAMP1*	*VAMP1*	*VAMP1*	*VAMP1*
**VCAN**	**VCAN**	**VCAN**	**VCAN**
**WNT5A**	**WNT5A**	**WNT5A**	**WNT5A**
*WNT10B*	*WNT10B*	*WNT10B*	*WNT10B*

Over-expressed genes are bold; Under expressed genes are italic. Genes highlighted in grey with underline are Markov blanket genes for the corresponding tumor grade.

In addition to Markov Blanket genes, Genie also predicted genes that are closed associated with Markov Blanket genes. For Pilocytic, P4HB, PTAFR,RASGRF1, SPARC,HLAF, PROS1, DCTN1,TGFB1, ZFP36L2, CDKN2D, VCAN, BCL2L2, SOX4, ODC1, CCND2; for Diffuse, IFI16, DPYSL3, PRKCG,HSD17B10, CD44, PLOD2, APBB1,TAGLN2, IDH3A, SSR2, HLAA, NONO; for Anaplastic, IGFBP5, SOD2, DCTN1, ANK3, SERINC3, WDR7, HLAE,SNRPE, NAMPT, CCND2, RPS19, CACNB1, DYNLT1; for Glioblastoma, PCNA, TPPP, CDH11, IGFBP2, FN1, KIF5C, LYPLA1,S100A10, PALLD genes were identified (see Figures 4–7).

A comparison of these Markov genes across each grade of Astrocytoma can be found in Table 3. None of the Markov blanket genes were common in all 4 stages of Astrocytomas. As Markov blanket genes, DUSP1 was common in Pilocytic and Diffuse tumors; EIF4A1 was common in Pilocytic, Diffuse and Anaplastic tumors; MARCKs was common in Diffuse and Anaplastic tumors and SERBP common in Anaplastic and Glioblastoma tumors.

### Predictive Analysis of Grade I Pilocytic Astrocytoma Markov Blanket Genes

Genie’s ‘learn parameters’ function was used to predict probabilities of Pilocytic tumor using DAG Bayesian network structure of 15 Markov blanket genes. Prediction analysis of the Grade I Markov genes by Genie showed that these 15 genes were was able to predict Pilocytic Astrocytoma tumor status consistently. The predictive ability of each individual gene is shown in the Figure 8. All 15 total Markov genes were used in the analysis, except in linear stepwise regression, which used only 10 significant predictor genes in the analysis. The prediction accuracy to distinguish normal or tumor was calculated using Bayesian Network Results using ROC Curve, Linear Regression (linR), Logistic Regression (logR), Cross Validation (CV) and Support Vector Machine (SVM). The accuracy of the predicting Pilocytic Tumor was very high, and varied between 79–88% (CV), 87–88 (linR), 100% (LogR), or 100% (SVM) (Table 4).

**Table 4:** Pilocytic Astrocytoma prediction analysis summary.

Type of Prediction Analysis	Case Counts (No Tumor/Tumor)	Predictability
**Bayesian Network Results using ROC Curve**	13/30	1.000 AUC (.000 sig.)
**Linear Regression (15 genes together)**	13/30	.873 (aR square) (.000 sig.)
**Linear Regression (stepwise) (10 gene model)**	13/30	.880 (aR square) (.000 sig.)
**Logistic Regression (discretized expression)**	13/30	Perfect fit detected
**Logistic Regression (raw expression)**	6/19	Perfect fit detected
**Cross Validation (discretized expression)**	13/30	79.1%
**Cross Validation (raw expression)**	6/19	88%
**Support Vector Machine (SVM)**	6/19	100%

**Figure 12:**
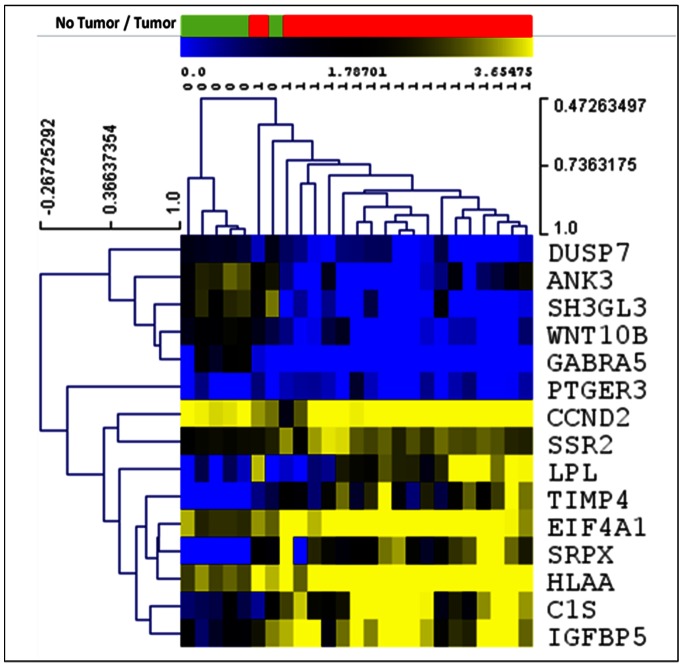
Hierarchical clustering of Pilocytic Astrocytoma Markov genes using raw expression values of Rickman study only. No Tumor/Tumor Bar: Red bar areas represent samples without tumors and green bar areas represent tumor cases. Expression: Blue squares represent underexpression; yellow squares represent overexpression.

Results of hierarchical clustering using expression data from our Markov genes are shown in Figure 12. Hierarchical clustering (Pearson distance, average linkage) of the 15-gene signature expression pattern shown green as normal control and red as Pilocytic tumor cases revealed that 100% of samples were distinguished as Pilocytic tumors.

### Predictive Analysis of Grade II Diffuse Astrocytoma Markov Blanket Genes

**Figure 13:**
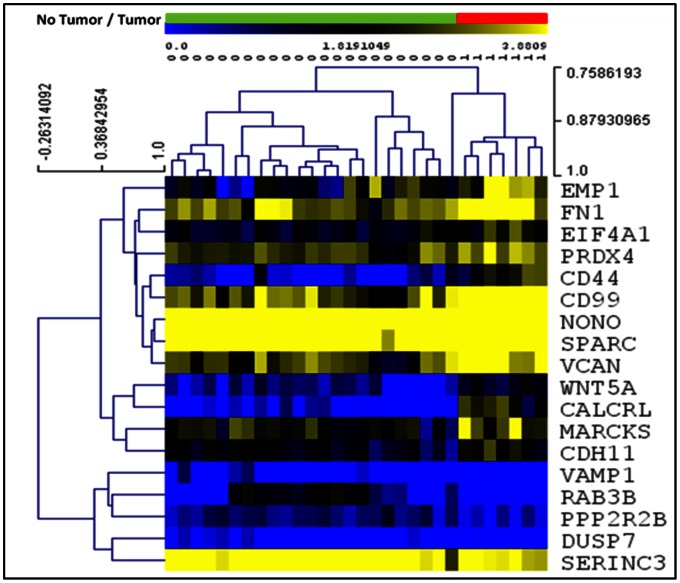
Hierarchical clustering of Diffuse Astrocytoma Markov genes using raw expression values of Sun Study Only. No Tumor/Tumor Bar: Green bar areas represent samples without tumors and red bar areas represent tumor cases. Expression: Blue squares represent underexpression; yellow squares represent overexpression.

Prediction analysis of the 18 Grade II Markov genes showed that our set of 18 genes was able to predict tumor status consistently when using logistic regression, cross validation and SVM analysis. Linear regression seemed to predict that a signature of 10 genes would predict tumor status just as well as our 18 total genes, though each gene set only predicted approximately 21% of the tumor status’s variability in either case (Table 5). Results of hierarchical clustering using expression data from our Markov genes can be found in Figure 13 and all 5 tumors were distinguished from normal control cases. We did not conduct further analysis of these same Markov genes for risk analysis of developing diffuse tumor because of a limited sample size.

**Table 5:** Diffuse Astrocytoma prediction analysis summary.

Type of Prediction Analysis	Case Counts(No Tumor/Tumor)	Predictability
**Bayesian Network Results using ROC Curve**	36/14	1.000 AUC (.000 sig.)
**Linear Regression (18 genes together)**	36/14	.207 (aR square) (.092 sig.)
**Linear Regression (stepwise) (10 gene model)**	36/14	.880 (aR square) (.028 sig.)
**Logistic Regression (discretized expression)**	36/14	88% (1.000 sig.)
**Logistic Regression (raw expression)**	23/8	Perfect fit detected
**Cross Validation (discretized expression)**	36/14	70%
**Cross Validation (raw expression)**	23/7	80%
**Support Vector Machine (SVM)**	23/7	100%

All 18 total Markov genes were used in the analysis, except in linear stepwise regression, which used only 10 significant predictor genes in the analysis.

### Predictive Analysis of Grade III Anaplastic Astrocytoma Markov Blanket Genes

**Figure 14:**
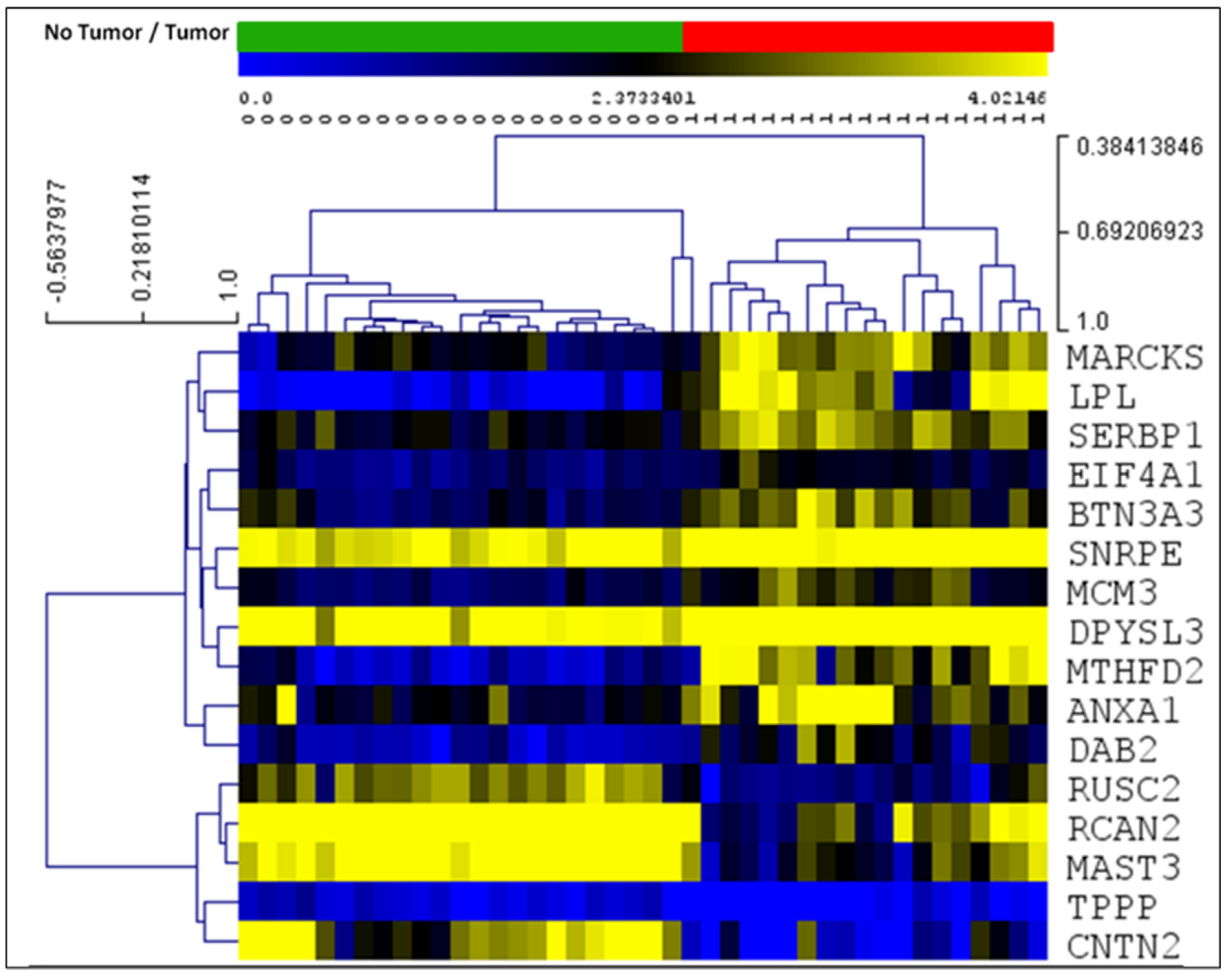
Hierarchical clustering of Anaplastic Astrocytoma Markov genes using raw expression values of Sun study only. No Tumor/Tumor Bar: Green bar areas represent samples without tumors and red bar areas represent tumor cases. Expression: Blue squares represent underexpression; Yellow squares represent overexpression.

Prediction analysis of the 18 Grade III Markov genes showed that our set of 18 genes was able to predict tumor status, especially when using logistic regression, raw expression in cross validation, and the SVM analysis. Linear regression seemed to again predict that a signature of much less than the total genes (10 of 18) would predict tumor status (Table 6). Results of hierarchical clustering using expression data showed Markov genes significantly distinguished tumors from normal samples and is shown in Figure 14. We did not conduct further analysis of these same Markov genes for risk analysis of developing anaplastic tumor because of a limited sample size.

**Table 6:** Anaplastic Astrocytoma prediction analysis summary.

Type of Prediction Analysis	Case Counts (No Tumor/Tumor)	Predictability
**Bayesian Network Results using ROC Curve**	34/23	1.000 AUC (.000 sig.)
**Linear Regression (18 genes together)**	34/23	.631 (aR square) (.000 sig.)
**Linear Regression (stepwise) (11 gene model)**	34/23	.645 (aR square) (.000 sig.)
**Logistic Regression (discretized expression)**	34/23	96.5% (1.000 sig.)
**Logistic Regression (raw expression)**	23/19	Perfect fit detected
**Cross Validation (discretized expression)**	34/23	84.2%
**Cross Validation (raw expression)**	23/19	100%
**Support Vector Machine (SVM)**	23/19	100%

All 18 total Markov genes were used in the analysis, except in linear stepwise regression, which used only 11 significant predictor genes in the analysis.

### Predictive Analysis of Glioblastoma Markov BLANKET CAUSAL GENES

In order to validate the key Markov blanket genes as causal/signature genes for possible Glioblastoma targets or biomarkers, it is necessary to analyze its predictive capability to distinguish between normal brain and tumor samples. Genie’s ‘learn parameters’ function analysis of the 10 Grade IV Markov genes showed that the set of 10 genes was able to consistently predict between non-tumor and tumor cases (Figure 11). Assessment of increased lifetime risk of development of glioblastoma due to deregulation of our Markov genes showed that differential expression of all 10 of our genes at once increased your lifetime risk of brain tumor development to 85.90%. In contrast, differential expression of two separate sets of 10 genes found outside the Markov blanket in our Bayesian network increased lifetime risk of brain tumor development to 2.61% and 0.98% respectively (Table 7).

**Table 7:** Risk associated with Markov genes vs. non-Markov genes in Glioblastoma Multiforme.

Gene Set	Risk
**Normal Gene Set**	0.61
**Markov Differentially Expressed (COL4A1, CD99, ANXA2, MPP2, EGFR, CDK4, BTF3, RAB31, TOP2A, SERBP1)**	85.90
**D-Connected Differentially Expressed (KIF5C, PALLD, S100A10, TPPP, PCNA, FN1, IGFBP2, CDH11, LYPLA1)**	2.61
**D-Separated Differentially Expressed (PKP4, SYNCRIP, CALCRL, GABRA5, MOBP, PLOD2, WNT5A, C1S, RPS2, FCHO1)**	0.98

Normal Gene Set Risk represents the SEER calculated 0.61% chance of a person developing a brain tumor in their lifetime.

### Interactions between Markov Blanket Genes and their Impact on the Risk of Developing Glioblastoma

We examined individual Markov blanket gene effect as well as joint effects of interaction between Markov Blanket genes on the lifetime risk of developing glioblastoma. Table 8 shows the estimated lifetime risk of developing glioblastoma from changes in the expression of the single Markov Blanket gene, a pair of Markov Blanket gene or multiple Markov Blanket genes. The estimated lifetime risk of developing breast cancer from altered expression of a single gene such as, COL4A1, CD99, ANXA2, MPP2, EGFR, CDK4, BTF3, RAB31, TOP2A or SERBP1 was 0.76, 1.18, 1.57, 1.62, 1.62, 1.85, 1.91, 0.73, 1.96 or 0.63%, respectively compared to 0.61% risk of developing glioblastoma in normal population. Joint effects of a pair, three or several Markov Blanket genes on the probability of increasing risk of developing Glioblastoma ranged from less than additive to greater than multiplicative. For example, joint effects of changes in the expression of different combination of a pair of Markov blanket genes or three Markov blanket genes increased risk for developing glioblastoma ranging from 0.73 to 5.84% and 1.46 to 4.47, respectively. The glioblastoma risk estimates were dramatically increased with joint effects of 4 or more than 4 Markov Blanket genes. Joint interactions between 4, 5, 6, 7, 8, 9 or 10 Markov blanket genes produced 9, 13, 20.9, 26.7, 52.8, 53.2, 78.1 or 85.9% increase, respectively, in lifetime risk of developing glioblastoma compared to normal population (0.61%). Whereas modified expression of two separate sets of 10 genes found outside the Markov Blanket in our Bayesian network only increased lifetime risk of brain tumor development to 2.61% and 0.98%, respectively compared to 0.61% in normal population.

**Table 8:** Impact of Interactions between Markov Blanket genes on the risk of developing glioblastoma.

Expressed Genes	COL4A1	COL4A1EGFR	COL4A1 BTF3	COL4A1 MPP2	COL4A1 RAB31	COL4A1 CDK4	COL4A1 CD99	COL4A1 ANXA2	COL4A1 TOP2A	COL4A1 SERBP1	COL4A1 EGFR BTF3	COL4A1 EGFR MPP2
Risk	0.76	1.46	1.94	2	2	1.05	2.32	0.76	2.37	0.76	3.67	3.78
Expressed Genes	COL4A1 EGFR RAB31	COL4A1 EGFR CDK4	COL4A1 EGFR CD99	COL4A1 EGFR ANXA2	COL4A1 EGFR TOP2A	COL4A1 EGFR SERBP1	COL4A1 EGFR BTF3 MPP2	COL4A1 EGFR BTF3 MPP2 RAB31	COL4A1_ EGFR_ BTF3_ MPP2_ RAB31_ CDK4	COL4A1_ EGFR_ BTF3_ MPP2_ RAB31_ CDK4_ CD99	COL4A1_ EGFR_ BTF3_ MPP2_ RAB31_ CDK4_ CD99_ ANXA2	COL4A1_ EGFR_ BTF3_ MPP2_ RAB31_ CDK4_ CD99_ ANXA2_ TOP2A
Risk	3.77	2.01	4.37	1.46	4.47	1.46	9.13	20.9	26.7	52.8	53.2	78.1
Expressed Genes	COL4A1_ BTF3_ MPP2_ RAB31_ CDK4_ CD99_ ANXA2_ TOP2A_ SERBP1	COL4A1_ EGFR_ MPP2_ RAB31_ CDK4_ CD99_ ANXA2_ TOP2A_ SERBP1	COL4A1_ EGFR_ BTF3_ RAB31_ CDK4_ CD99_ ANXA2_ TOP2A_ SERBP1	COL4A1_ EGFR_ BTF3_ MPP2_ CDK4_ CD99_ ANXA2_ TOP2A_ SERBP1	COL4A1_ EGFR_ BTF3_ MPP2_ RAB31_ CD99_ ANXA2_ TOP2A_ SERBP1	COL4A1_ EGFR_ BTF3_ MPP2_ RAB31_ CDK4_ ANXA2_ TOP2A_ SERBP1	COL4A1_ EGFR_ BTF3_ MPP2_ RAB31_ CDK4_ CD99_ TOP2A_ SERBP1	COL4A1_ EGFR_ BTF3_ MPP2_ RAB31_ CDK4_ CD99_ ANXA2_ SERBP1	COL4A1_ EGFR_ BTF3_ MPP2_ RAB31_ CDK4_ CD99_ ANXA2_ TOP2A_	COL4A1_ EGFR_ BTF3_ MPP2_ RAB31_CDK4_ CD99_ ANXA2_ TOP2A_ SERBP1		
Risk	76	70.4	69.8	69.8	81.5	66.2	85.7	53.2	78.1	85.9		

Normal Gene Set Risk represents the SEER calculated 0.61% chance of a person developing a brain tumor in their lifetime.

### Validation of the 10 Key Markov Blanket Causal/signature Glioblastoma Genes

All 10 total Markov genes were used in the analysis, except in linear stepwise regression, which used only 6 significant predictor genes in the analysis. Prediction analysis of the Grade I Markov genes showed that these 10 genes were was able to predict Pilocytic Astrocytoma tumor status consistently in all analyses (Table 9). The accuracy of the predicting Glioblastoma Tumor compared to the normal samples was very high, and varied between 63–64 (linR), 84–100% (CV), 96–100% (LogR), or 100% (SVM) (Table 9). In summary, this result demonstrates that the 10 gene signature is a good predictor of Glioblastoma vs. normal brain.

**Table 9:** Glioblastoma Multiforme prediction analysis summary.

Type of Prediction Analysis	Case Counts (No Tumor/Tumor)	Predictability
**Bayesian Network Results using ROC Curve**	34/23	1.000 AUC (.000 sig.)
**Linear Regression (18 genes together)**	34/23	.631 (aR square) (.000 sig.)
**Linear Regression (stepwise) (11 gene model)**	34/23	.645 (aR square) (.000 sig.)
**Logistic Regression (discretized expression)**	34/23	96.5% (1.000 sig.)
**Logistic Regression (raw expression)**	23/19	Perfect fit detected
**Cross Validation (discretized expression)**	34/23	84.2%
**Cross Validation (raw expression)**	23/19	100%
**Support Vector Machine (SVM)**	23/19	100%

All 10 total Markov genes were used in the analysis, except in linear stepwise regression, which used only 6 significant predictor genes in the analysis.

**Figure 15:**
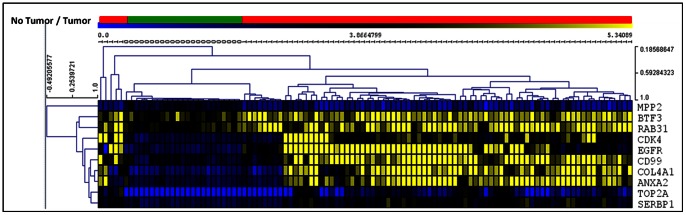
Hierarchical clustering of Glioblastoma Multiforme genes using raw expression values of Sun study only. No Tumor/Tumor Bar: Green bar areas represent samples without tumors and red bar areas represent tumor cases. Expression: Darker squares represent underexpression; Lighter squares represent overexpression.

Results of hierarchical clustering using expression data from our Markov genes are shown in Figure 15. Hierarchical clustering (Pearson distance, average linkage) of the 15-gene signature expression pattern shown green as normal control and red as, Pilocytic tumor cases distinguished most of the Glioblastoma tumors and normal cases.

### Literature Based Validation of 10 Key Markov Blanket Causal/signature Glioblastoma Genes Using Empirical Pathways Finders

**Figure 16:**
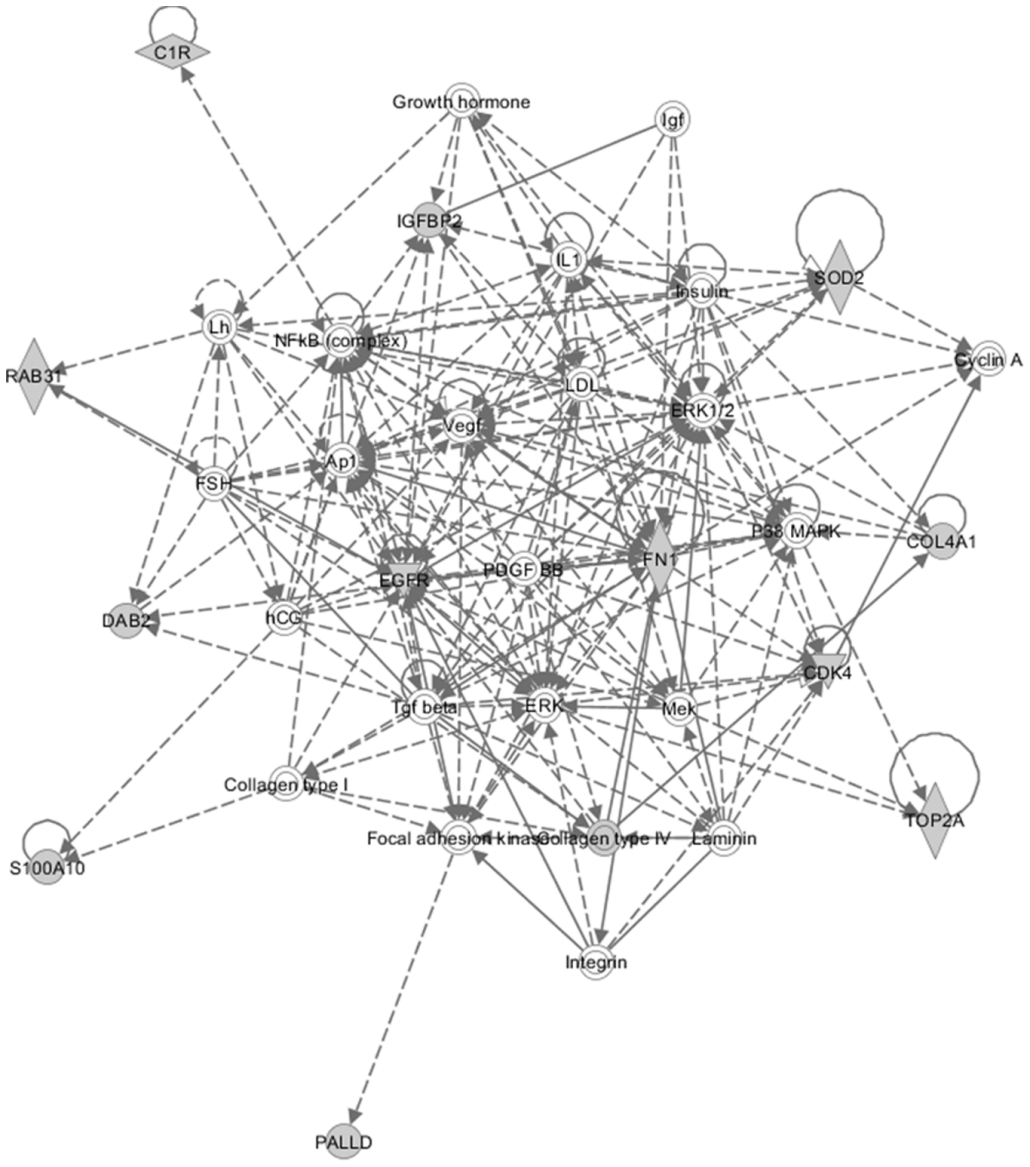
Top IPA Grade IV Glioblastoma Markov genes network. IPA defined the network as related to ‘cancer, neurological disease, and cellular movement’. Shaded genes represent our Markov genes. Non-shaded molecules were added by IPA during the analyses.

**Figure 17:**
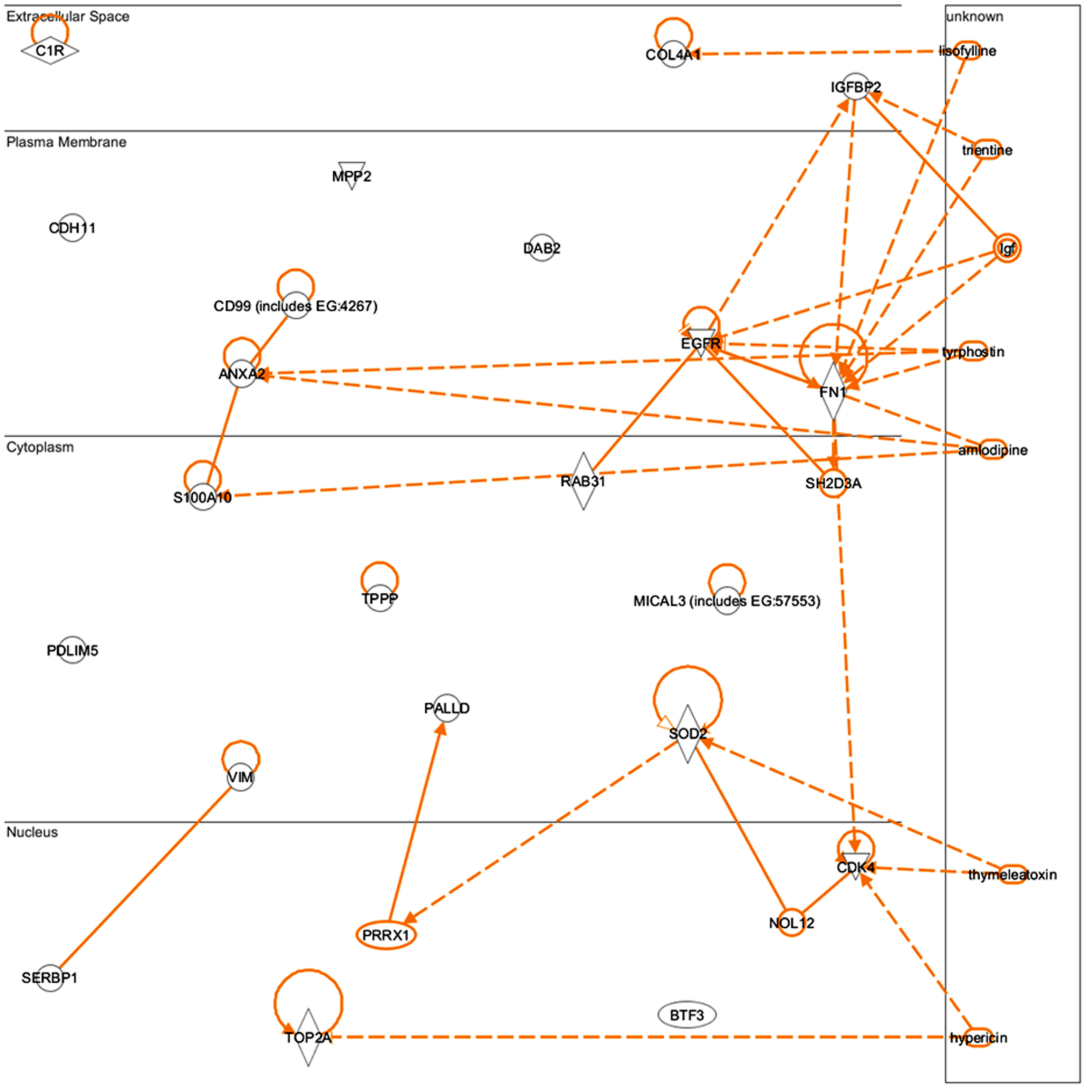
IPA connections of Grade IV Glioblastoma Markov genes. Direct interactions between genes (genes/gene products make direct physical contact with each other) are represented by solid lines. Indirect interactions (genes/gene products do not make direct physical contact with each other but instead may influence each other through some intermediate factor) are represented as dotted lines.

**Figure 18:**
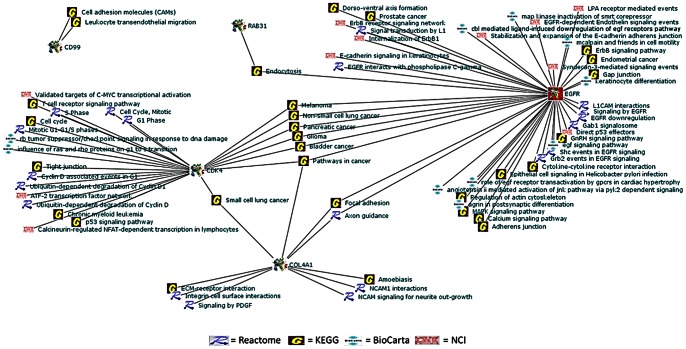
Results of PathJam analysis of Grade 4 Astrocytoma Markov genes. Arcs represent interactions between genes and pathways. Databases for curated pathways and gene ontology terms are identified by their respective emblems.

Research supporting the potential involvement and importance of all 10 genes in development of glioblastoma was found in the literature. For a full list of the biological pathways and gene ontology terms associated with each gene please see Table 10. The investigation using IPA’s Path Explorer produced a network of genes showing empirical evidence of interaction among our 10 Glioblastoma Markov blanket genes. IPA Core Analysis of these same Markov genes added genes and molecules such as NFkβ, ERK, MAPK, VEGF, growth hormone and collagen to produce a network whose top biological functions are cancer, neurological disease, and cellular movement (Figure 16). IPA connections of Grade IV Glioblastoma Markov genes reveled direct interactions between CD99 and ANXA2, and EGFR and RAB31 genes (Figure 17). Our analysis of these same Markov genes using PathJam found that three of the 10 genes in particular seemed to be potential ‘hubs of activity’ and had functions that they shared (Figure 18). These genes, EGFR, COL4A1, and CDK4 all shared the ‘pathways to cancer’ annotation; and EGFR and COL4A1 were shown to be involved specific cancers such as glioma, melanoma, lung cancer, bladder cancer, and pancreatic cancer. Additionally, COL4A1 and EGFR shared involvement in axon guidance and focal adhesion.

**Table 10:** Characteristics of Grade IV Glioblastoma Multiforme Markov blanket genes.

Gene Symbol/Name	Genomic Location/Cellular Localization	Function	Cancer/Disease Link
**COL4A1/Collagen, type IV, alpha 1**	13q34/extracellular matrix	Inhibits angiogenesis and tumor formation	Are upregulated in malignant and metastatic brain tumors [32]
**EGFR/Epidermal growth factor receptor**	7p12/membrane	Growth factor	Consistently linked to development of glioblastoma [25]; Has been linked to glioma tumor invasiveness, proliferation, and angiogenesis [33], [34]; Mutations have been found in EGFR in primary glioblastomas [35] and have been linked to poor prognosis in GBM [36]. Its signaling has been shown to cooperate with loss of tumor suppressor gene functions in promotion of gliomagenesis
**BTF3/Basic transcription factor 3**	5q13.2/nucleus	Transcription factor	Found to be highly expressed in glioblastoma multiforme [37]; regulates tumor-associated genes in pancreatic cancer cells [38]
**MPP2/Membrane protein, palmitoylated 2**	17q12-q21/membrane	Tumor suppressor; Coupling of cytoskeleton to cell membrane	Contributes to cell proliferation and resistance in cisplatin treatment in medulloblastoma cells [39]
**RAB31/Member RAS oncogene family**	18q11.3/membrane	Vesicle and granule targeting	May have role in regulating EGFR in astrocyte development and oncogenesis [40]; associated with survival in glioblastoma [41]
**CDK4/Cyclin-dependent kinase 4**	12q14/cytoplasm	Cell cycle regulation; inhibits RB protein family members	Known target of glioblastoma anticancer therapy [42]; thought to be a driver mutation gene in glioblastoma [43]
**CD99/CD99 molecule**	Xp22.32/membrane	Leukocyte migration, T-cell adhesion, protein transport, and T-cell death	May act as an oncosuppressor in osteosarcoma; Is a useful marker for diagnosis of brain tumor types [44]
**ANXA2/Annexin A2**	15q22.2/Extracellular space, extracellular matrix, membrane	Regulation of cell growth	Involved in migration of neural stem cells to glioma sites [45]; potentially involved in glioma invasion [46]
**TOP2A/Topoisomerase (DNA) II alpha 170kDa**	17q21-q22/Cytosplasm, nucleus, nucleoplasm	Resolves topological problems in genomic DNA resulting from replication, transcription and repair	Is target of several anticancer agents; mutations in this gene have been associated with development of drug resistance; common significantly altered gene in cancer [47]; May be involved in network of genes controlling cell cycle regulation in glioblastoma [48]; very high copy number gain in glioblastoma [49]
**SERBP1/SERPINE1 mRNA binding protein 1**	1p31/Cytoplasm, nucleus	Regulation of mRNA stability	Significantly overexpressed in ovarian cancer, especially in advanced disease [50]

## Discussion

Our study produced several major novel findings, including identification of a list of top over- and under-expressed genes among 10 sub-studies on astrocytoma, identification of several key signature genes important to the development of both low and high grade astrocytomas, identification of important signaling pathways in astrocytic stage 4 tumors, and identification of possible mechanisms which may explain the genes and pathways identified as important to development of glioblastoma.

Through meta-analysis of 10 sub-studies which compared normal tissue to astrocytomas, a set of 646 genes which were differentially expressed in the majority of these studies was identified. Many of the genes identified through this meta-analysis have in fact been implicated in development of astrocytoma including EGFR (amplification occurs in ∼40% of primary glioblastomas [23], [24], HIF-1α, MYC, WNT5A, and IDH3A. Enrichment analysis of the 646 genes using FuncAssociate identified several processes associated with these genes, many of which are related to nervous system, developmental, and tumor promoting processes. Ingenuity Pathway Analysis also produced a list of processes that are significantly associated with these genes, including two pathways which have previously been linked to development of astrocytomas [1. ‘WNT/beta-Catenin Signaling’ (Genes from our set in pathway: CD44, CDH2, DVL3, LRP1, MYC, SOX4, SOX9, SOX13, TCF3, TCF4, TLE3, WNT5A) and 2. ‘mTOR Signaling’ (Genes: EIF3B, EIF3E, EIF3F, EIF4A1, HIF1A, PRKD1, RHOC, RND2, RND3)] and two pathways associated with brain tumor development [1. ‘Glioma Invasiveness Signaling’ (Genes: CD44, F2R, ITGAV, MMP9, RHOC, RND2, RND3, TIMP3, TIMP4) and ‘Glioblastoma Multiforme Signaling’ (Genes: CDK6, CDKN1A, EGFR, ITPR2, MYC, RHOC, RND2, RND3, TCF3, WNT5A)].

In order to narrow our large set of genes to a few genes which could be most influential to development of astrocytomas, we performed reverse engineering of our gene list using Bayesian network analysis. Four networks of genes were produced, one for each grade of Astrocytoma. Genes found to be most influential to development of the highest grade of astrocytoma, Glioblastoma multiforme (GBM) were: COL4A1, EGFR, BTF3, MPP2, RAB31, CDK4, CD99, ANXA2, TOP2A, and SERBP1. All of these genes were up-regulated, except MPP2 (down regulated). Tumor status of 10 Markov blanket genes predicted by Bayesian network analysis was validated using linear regression, logistic regression, cross validation, hierarchal clustering and support vector machine (SVM) analysis. These 10 genes were able to predict tumor status with high accuracy by all methods. Analysis of gene-gene interactions revealed that joint effects of changes in the expression of different combination of a pair of Markov blanket genes or three Markov blanket genes increase risk for developing glioblastoma ranging from 0.73 to 5.84% and 1.46 to 4.47, respectively. The glioblastoma risk estimates are dramatically increased with joint effects of 4 or more than 4 Markov Blanket genes. Joint interactions between 4, 5, 6, 7, 8, 9 or 10 Markov blanket genes produced 9, 13, 20.9, 26.7, 52.8, 53.2, 78.1 or 85.9% increase, respectively, in lifetime risk of developing glioblastoma compared to normal population (0.61%). The differential expression of two separate sets of 10 genes found outside the Markov blanket in our Bayesian network only increase lifetime risk of brain tumor development to 2.61% and 0.98%, respectively compared to 0.61% in normal population. In summary, it appears that differential expression of COL4A1, EGFR, BTF3, MPP2, RAB31, CDK4, CD99, ANXA2, TOP2A, and SERBP1 genes may be required for the development of glioblastoma (GBM), the most common type of malignant brain tumor.

To investigate the biological mechanisms of our set of significant Grade IV network genes we used biological databases such as The Human Gene Compendium’s Gene Cards, PubMed, the Information Hyperlinked over Proteins (iHOP) Database, and the Glioblastoma Multiforme Database (GBMBase). Major patterns in these tumors include components of the Ras-MAPK and PI3K-AKT-mTOR signaling pathways being affected in the plurality (88%; 80 of 91) of malignant gliomas and disruption of the p53 and RB tumor suppressor networks also occurring in a high proportion of glioblastomas: 87% (79 of 91) and 78% (71 of 91), respectively [25]. Our findings revealed that most of the glioblastoma Markov Signature genes are up-regulated in malignant and metastatic brain tumors, linked to the glioma tumor development, invasiveness, proliferation, and angiogenesis [34]–[47]. Their signaling has been shown to cooperate with tumor-associated gene functions involved in oncogenesis. Markov signature genes identified in this study interact with NFkβ, ERK, MAPK, VEGF, growth hormone and collagen to produce a network whose top biological functions are cancer, neurological disease, and cellular movement. Three of the 10 Markov causal genes - EGFR, COL4A1, and CDK4, in particular seemed to be potential ‘hubs of activity’. These three genes share the ‘pathways to cancer’ annotation.

There are several strengths and limitations involved with our analyses. Several characteristics of microarray expression studies must be considered. First, expression levels of many genes differ among individuals and thus gene expression can be analyzed like other quantitative phenotypes such as height and blood glucose levels. This allowed us to separate each gene into expression categories of over-, median, and under-expression. Expression changes can also reflect many types of alterations significant to tumor development, including chromosomal translocations and epigenetic alterations. Additionally, several studies have established causal links between differential gene expression and complex disease risk and thus identification of over- and under-expressed genes in tumor tissue compared to normal tissue could provide important clues to the development of tumors. Furthermore, it has also been shown that genes with similar expression patterns form complexes and/or pathways that are part of regulatory circuits that may lead to tumors and other diseases, lending support to the validity of our pathway analyses. Our meta-analysis, which took the top 600 over- and top 600 under-expressed genes from a set of studies, should also have produced the most important differentially expressed genes across all astrocytic tumors. Analysis that shows 143 genes in GBM are expressed on average at 10-fold higher levels than normal tissue confirms that the most highly expressed genes in GBM were considered in our analysis [26].

There are also several limitations of expression values. Foremost are the discrepancies between protein and mRNA levels in studies correlating their expression, a clear sign that interactions outside the classical DNA to mRNA to protein pathway are taking place inside the cell. Additionally, it has been shown that known genes may not necessarily be differentially expressed in diseases due to the ability of mutations in the coding regions of genes and post-translational modifications affecting gene function without affecting its expression level. However, our approach of meta-analysis and focusing on networks of genes rather than single genes may lessen the effect of missing important genes (i.e. while one gene in a pathway may not be expressed, another may). Finally, only looking at mean expression changes of genes could lead to incorrect conclusions about the involvement of a pathway in a disease condition, and so as suggested by de la Fuente 2010, co-expression of genes should also be considered [27].

Limitations of enrichment analysis in general apply [28] to our analysis, including: a) incomplete annotation databases as a result of only a subset of known genes being functionally annotated; b) annotation databases may not be completely updated with all literature results; c) some annotation assignments may be erroneous, especially those which are electronically inferred; d) singling out the most important processes for genes involved in several biological processes is limited. This can be overcome by looking at the gene in context of other over- and/or under-expressed genes however; and e) annotation bias due to some biological processes being studied in more detail than others (e.g. proliferation).

Another limitation of our approach is that because our set of significant genes was chosen through meta-analysis of micro-array studies that used differing platforms and differing gene totals per study, we were unable to input a set of genes as for our ‘total gene universe’ in our gene enrichment analyses. This limited us to choosing the entire genome as our universe of comparison genes for the enrichment analyses. However, 4 of the 9 studies contained 18,800+ genes and one other study contained 14,584 genes, making it likely that most of our selected significant genes represent most of the appropriate over- and/or under-expressed genes in astrocytoma.

Reverse network engineering methods have evolved greatly over the past decade, with recent reports lending credibility to their ability to correctly predict biological interactions [29], [30]. However, limitations associated with their use must be considered. In particular, static Bayesian networks cannot contain feedback loops, due to the steady state nature of the data. Thus, a characteristic common to biological systems was not considered in our network. Also, because Bayesian networks model probabilistic dependencies among variables and not causality, we cannot conclusively say that the parents of a node are direct causes of its behavior [4]. A causal link can be inferred however, if the Causal Markov Condition holds true. Simply, this condition states that any node in a Bayesian network is conditionally independent of its non-descendants, given its parents; and, a node is conditionally independent of the entire network, given its Markov blanket. A strength of our approach is the exploration of gene networks in tumors without a priori genetic interaction networks being assumed. This has been mentioned as a limitation of previous work on gene networks in gliomas [31]. Incorporation of biological evidence that directs our Bayesian network search could serve to strengthen our approach in the future however.

Finally, limitations concerning the data used in our study must be considered. For example, our inability to separate pediatric astrocytomas from adult astrocytomas, secondary glioblastomas from secondary glioblastomas, and male vs. female cases does limit the extent to which we can draw conclusions from our data. The possibility that ‘a fraction of GBMs designated as primary tumors may follow a sequence of genetic events similar to that of secondary lesions but not come to clinical attention until malignant progression to a GBM has occurred’, lessens the concern of dividing types of glioblastomas however. Additionally, our method could be considered non-biased in this respect, as it does not pre-condition results based on priors, thus allowing for a search which may provide key genes across all hypothesized glioblastoma subtypes.

In summary, the major novel findings which emerged from this study are that modified expression of Markov Blanket COL4A1, EGFR, BTF3, MPP2, RAB31, CDK4, CD99, ANXA2, TOP2A, and SERBP1 genes are associated with the development of glioblastoma, a highest form of astrocytoma. Modified expression of these 10 Markov Blanket genes increases lifetime risk of developing glioblastoma. Analysis of gene-gene interactions revealed that the glioblastoma risk estimates were dramatically increased with joint effects of 4 or more than 4 genes. Joint effects of 4, 5, 6, 7, 8, 9 or 10 Markov Blanket genes on lifetime risk of developing glioblastoma were 9, 13, 20.9, 26.7, 52.8, 53.2, 78.1 or 85.9%, respectively. Findings of our study have major implications in understanding the development of astrocytoma. Findings of this study not only identify key important molecular determinants and a new paradigm critical to the development of astrocytoma; it also provides important information for the design of new gene therapy targeted for the prevention and treatment of brain cancer. Though these molecules could be causally linked to astrocytoma, further detailed analysis is necessary. Experiments involving system perturbations of these genes (e.g. gene knockout experiments) are needed to establish directionality in our network and to provide validity of our findings. Further studies are needed to define the mechanism of action of these genes, and validation of these ‘key genes’ by prospective studies could potentially lead to useful tools for early detection and novel therapeutic options for glioblastoma multiforme, and other astrocytomas.

## Supporting Information

File S1 **Tables S1–S3.**
(DOCX)
