# Supplementary material for: Reverse Engineering of Modified Genes by Bayesian Network Analysis Defines Molecular Determinants Critical to the Development of Glioblastoma
Source: PLoS One. 2013 May 30;8(5):e64140. doi: 10.1371/journal.pone.0064140 (PMC3667850; doi:10.1371/journal.pone.0064140)
Supplement: File S1 — Tables S1–S3. (DOCX) [file pone.0064140.s001.docx]

**Combined Supporting Information files**

**Table S1. Significantly over-expressed gene list from Oncomine meta-analysis of normal vs. astrocytoma studies.** *Genes are ranked by significance in Oncomine. **Genes were considered significant in the meta-analysis, and chosen for further analysis, if they were found in at least in at least 7 of 10 meta-analyzed studies.

| Gene Significance Rank* | Gene Symbol | Studies  (of 10)** | Gene Significance Rank* | Gene Symbol | Studies  (of 10)** |
| --- | --- | --- | --- | --- | --- |
|  |  |  |  |  |  |
| 1 | SYNCRIP | 9 | **187** | PLAU | 10 |
| 2 | RAB31 | 10 | **188** | HLA-DRB1 | 10 |
| 3 | HLA-G | 8 | **189** | SERPING1 | 10 |
| 4 | FN1 | 10 | **190** | RB1 | 8 |
| 5 | HLA-J | 7 | **191** | NFKB1A | 10 |
| 6 | SOX4 | 10 | **192** | PTTG1IP | 10 |
| 7 | C1R | 10 | **193** | RPL12 | 9 |
| 8 | SNRPG | 9 | **194** | CXCR4 | 9 |
| 9 | NAMPT | 9 | **195** | FYB | 10 |
| 10 | TIMP3 | 10 | **196** | ITGAV | 10 |
| 11 | GNB2L1 | 10 | **197** | ISG15 | 10 |
| 12 | IGFBP7 | 9 | **198** | GALNT1 | 10 |
| 13 | HIF1A | 10 | **199** | SEC11A | 8 |
| 14 | RPLP0 | 9 | **200** | EMP3 | 10 |
| 15 | ANXA2 | 10 | **201** | IFI44 | 8 |
| 16 | LPL | 10 | **202** | CAV1 | 10 |
| 17 | ID3 | 7 | **203** | RIT1 | 10 |
| 18 | PRPF40A | 9 | **204** | ATP6V0E1 | 9 |
| 19 | MARCKS | 10 | **205** | RBMS1 | 9 |
| 20 | P4HB | 10 | **206** | SMAD1 | 10 |
| 21 | PALLD | 8 | **207** | LPCAT1 | 8 |
| 22 | COL4A1 | 10 | **208** | FOXD1 | 9 |
| 23 | PRDX4 | 10 | **209** | EIF4A3 | 10 |
| 24 | SERBP1 | 9 | **210** | AEBP1 | 10 |
| 25 | IFI16 | 10 | **211** | MYCBP | 10 |
| 26 | PROS1 | 10 | **212** | DDX39 | 8 |
| 27 | DPYSL3 | 10 | **213** | CTSO | 10 |
| 28 | IDH1 | 8 | **214** | IL13RA1 | 10 |
| 29 | DYNLT1 | 9 | **215** | CFI | 9 |
| 30 | NONO | 10 | **216** | TFPI | 10 |
| 31 | SOD2 | 10 | **217** | G3BP1 | 10 |
| 32 | SPARC | 10 | **218** | DAG1 | 10 |
| 33 | TIMP4 | 10 | **219** | CKS2 | 10 |
| 34 | DBI | 9 | **220** | PABPC1 | 10 |
| 35 | CD151 | 10 | **221** | AK2 | 9 |
| 36 | SNRPE | 9 | **222** | PTPN12 | 10 |
| 37 | LYPLA1 | 9 | **223** | ILF2 | 10 |
| 38 | ZFP36L2 | 10 | **224** | SP3 | 10 |
| 39 | HLA-F | 10 | **225** | SSR4 | 10 |
| 40 | HLA-B | 8 | **226** | SOX6 | 7 |
| 41 | PTBP1 | 9 | **227** | LAPTM4B | 9 |
| 42 | PCNA | 10 | **228** | CDKN2C | 9 |
| 43 | COL1A2 | 10 | **229** | F13A1 | 10 |
| 44 | BTF3 | 9 | **230** | SP110 | 7 |
| 45 | WNT5A | 10 | **231** | TOP1 | 10 |
| 46 | CCND2 | 10 | **232** | ANXA5 | 10 |
| 47 | CD44 | 10 | **233** | MUC1 | 10 |
| 48 | EIF4A1 | 10 | **234** | VAT1 | 10 |
| 49 | CSDA | 10 | **235** | UBE2J1 | 9 |
| 50 | PDLIM5 | 9 | **236** | KCTD12 | 7 |
| 51 | HLA-E | 9 | **237** | INPPL1 | 10 |
| 52 | NNMT | 10 | **238** | TAP1 | 10 |
| 53 | CD99 | 10 | **239** | STAT1 | 10 |
| 54 | IGFBP5 | 10 | **240** | GBP1 | 10 |
| 55 | ANXA1 | 10 | **241** | PHLDA1 | 10 |
| 56 | CALCRL | 10 | **242** | COPB2 | 10 |
| 57 | IGFBP2 | 9 | **243** | DECR1 | 10 |
| 58 | EMP1 | 8 | **244** | COL4A2 | 10 |
| 59 | BTG1 | 10 | **245** | SNRNP200 | 8 |
| 60 | SRPX | 10 | **246** | SOX9 | 10 |
| 61 | SSR2 | 9 | **247** | KIF14 | 10 |
| 62 | PRRX1 | 8 | **248** | ACBD3 | 9 |
| 63 | TRAF3IP2 | 9 | **249** | DARS | 10 |
| 64 | CDK4 | 10 | **250** | TAPBP | 9 |
| 65 | EGFR | 10 | **251** | LYN | 10 |
| 66 | TOP2A | 10 | **252** | CHI3L2 | 10 |
| 67 | PTPRZ1 | 10 | **253** | BCL6 | 10 |
| 68 | SAT1 | 10 | **254** | ILF3 | 10 |
| 69 | ABCC3 | 9 | **255** | LMNB1 | 9 |
| 70 | WEE1 | 10 | **256** | TGIF1 | 10 |
| 71 | TAGN2 | 9 | **257** | NID1 | 10 |
| 72 | S100A10 | 10 | **258** | PPP4C | 10 |
| 73 | MCM3 | 10 | **259** | CANX | 10 |
| 74 | STAT3 | 10 | **260** | NMI | 10 |
| 75 | RPS2 | 9 | **261** | COL1A1 | 7 |
| 76 | BTN3A3 | 9 | **262** | SEC61G | 9 |
| 77 | MTHFD2 | 10 | **263** | TRIO | 10 |
| 78 | RPS19 | 10 | **264** | DNALI1 | 9 |
| 79 | VCAN | 10 | **265** | F2R | 10 |
| 80 | IRAK1 | 10 | **266** | PSMB9 | 9 |
| 81 | CDH11 | 10 | **267** | C1orf61 | 10 |
| 82 | RPL18A | 9 | **268** | CEBPD | 9 |
| 83 | DAB2 | 10 | **269** | SLC2A5 | 10 |
| 84 | TMX1 | 9 | **270** | TCF4 | 10 |
| 85 | PSMB8 | 7 | **271** | LSM7 | 8 |
| 86 | HLA-A | 10 | **272** | EDNRA | 10 |
| 87 | CD63 | 8 | **273** | PLXNB2 | 8 |
| 88 | HSD17B10 | 10 | **274** | SSR1 | 10 |
| 89 | DAP | 10 | **275** | SCP2 | 10 |
| 90 | UBE2L6 | 9 | **276** | SUPR1 | 9 |
| 91 | DFNA5 | 7 | **277** | PXDN | 10 |
| 92 | TGFB1 | 10 | **278** | OSMR | 9 |
| 93 | YBX1 | 8 | **279** | RNASE6 | 10 |
| 94 | C1S | 10 | **280** | IL1RAP | 9 |
| 95 | FCGBP | 10 | **281** | GLB1 | 10 |
| 96 | PYGL | 10 | **282** | SON | 10 |
| 97 | SHMT2 | 10 | **283** | LIMS1 | 10 |
| 98 | PLOD2 | 10 | **284** | NEDD4 | 10 |
| 99 | ODC1 | 10 | **285** | ZNF22 | 10 |
| 100 | VIM | 10 | **286** | WTAP | 10 |
| 101 | PSMA2 | 10 | **287** | RPL29 | 10 |
| 102 | LTF | 7 | **288** | EIF3B | 10 |
| 103 | IGFBP3 | 10 | **289** | TM9SF1 | 10 |
| 104 | ABCA1 | 8 | **290** | EIF3E | 10 |
| 105 | WDR1 | 8 | **291** | CD14 | 10 |
| 106 | NCK1 | 10 | **292** | TNX8 | 7 |
| 107 | B2M | 10 | **293** | ENTPD1 | 10 |
| 108 | KDELR2 | 10 | **294** | RBBP4 | 10 |
| 109 | CTSC | 10 | **295** | GPR56 | 8 |
| 110 | GNAS | 10 | **296** | STAB1 | 8 |
| 111 | CHI3L1 | 10 | **297** | BUD31 | 10 |
| 112 | SFRS3 | 10 | **298** | DTYMK | 10 |
| 113 | RAB13 | 8 | **299** | LRP10 | 8 |
| 114 | ADAM9 | 10 | **300** | TRIB2 | 10 |
| 115 | TNC | 10 | **301** | GNA12 | 9 |
| 116 | HNMT | 9 | **302** | RBMX | 10 |
| 117 | CALD1 | 10 | **303** | HEBP2 | 9 |
| 118 | APOC1 | 10 | **304** | SFRS1 | 9 |
| 119 | PSME2 | 10 | **305** | ARHGDIB | 10 |
| 120 | MCL1 | 10 | **306** | FLNA | 10 |
| 121 | PHB2 | 10 | **307** | VCAM1 | 10 |
| 122 | MYC | 10 | **308** | CSNK1A1 | 10 |
| 123 | RELA | 9 | **309** | PSMF1 | 10 |
| 124 | PLTP | 8 | **310** | PDIA6 | 9 |
| 125 | SOAT1 | 7 | **311** | GNS | 10 |
| 126 | NME4 | 10 | **312** | RUNX1 | 10 |
| 127 | RHOC | 10 | **313** | RPL17 | 8 |
| 128 | HLA-C | 7 | **314** | SMC5 | 9 |
| 129 | ZC3HAV1 | 9 | **315** | ETV1 | 10 |
| 130 | JAG1 | 10 | **316** | LAMC1 | 10 |
| 131 | CSRP2 | 10 | **317** | PCOLCE | 10 |
| 132 | BARD1 | 10 | **318** | RECQL | 10 |
| 133 | LAMA4 | 9 | **319** | VAMP8 | 8 |
| 134 | PRCP | 9 | **320** | ARHGEF6 | 10 |
| 135 | TIMP1 | 10 | **321** | BNIP2 | 10 |
| 136 | CBX3 | 10 | **322** | FADD | 10 |
| 137 | NME2 | 7 | **323** | HLA-DRA | 10 |
| 138 | CPNE3 | 8 | **324** | MDK | 9 |
| 139 | CD163 | 10 | **325** | GALNT10 | 9 |
| 140 | POLR2J | 10 | **326** | TNPO1 | 10 |
| 141 | CKAP4 | 10 | **327** | HMGN1 | 9 |
| 142 | PTN | 9 | **328** | EIF4EBP1 | 8 |
| 143 | LYPD1 | 9 | **329** | BTN3A2 | 9 |
| 144 | DLG5 | 9 | **330** | TMCO1 | 9 |
| 145 | LAPTM4A | 9 | **331** | KARS | 10 |
| 146 | ID4 | 10 | **332** | POSTN | 10 |
| 147 | WWTR1 | 9 | **333** | ELF1 | 10 |
| 148 | CALU | 9 | **334** | TPM2 | 10 |
| 149 | GBE1 | 10 | **335** | GTF3C2 | 10 |
| 150 | FYN | 10 | **336** | GBP2 | 9 |
| 151 | ERGIC3 | 9 | **337** | TP53 | 9 |
| 152 | BACH1 | 8 | **338** | GAS1 | 10 |
| 153 | SERPINE1 | 10 | **339** | CYFIP1 | 10 |
| 154 | POLD2 | 10 | **340** | JUN | 10 |
| 155 | AIF1 | 10 | **341** | RCN1 | 10 |
| 156 | FCGRT | 10 | **342** | HNRNPUL1 | 9 |
| 157 | H19 | 8 | **343** | HAT1 | 9 |
| 158 | H2AFV | 9 | **344** | MAPK7 | 10 |
| 159 | MAPRE1 | 10 | **345** | TRIM14 | 10 |
| 160 | KIAA0040 | 7 | **346** | UBE2C | 10 |
| 161 | TPR | 10 | **347** | MAPKAPK2 | 10 |
| 162 | RPL23 | 10 | **348** | SP100 | 10 |
| 163 | COL3A1 | 10 | **349** | SFPQ | 10 |
| 164 | DPY19L1 | 9 | **350** | FKBP5 | 10 |
| 165 | APOE | 10 | **351** | HLA-DMA | 10 |
| 166 | PDPN | 9 | **352** | H3F3A | 10 |
| 167 | GNG5 | 9 | **353** | NPM1 | 7 |
| 168 | NUP205 | 10 | **354** | NAGA | 7 |
| 169 | SSRP1 | 10 | **355** | CTGF | 10 |
| 170 | HLA-DQB1 | 10 | **356** | GUSB | 10 |
| 171 | LAMB2 | 10 | **357** | RPA1 | 10 |
| 172 | LHFPL2 | 10 | **358** | MPZL1 | 9 |
| 173 | BAT1 | 8 | **359** | LY96 | 8 |
| 174 | OBSL1 | 9 | **360** | HLA-DPB1 | 10 |
| 175 | CNN3 | 10 | **361** | AIMP2 | 9 |
| 176 | PPIB | 10 | **362** | ARPC1B | 10 |
| 177 | STK17A | 9 | **363** | EEF1G | 10 |
| 178 | ACLY | 10 | **364** | SH3BP2 | 10 |
| 179 | FCGR1A | 9 | **365** | UBA7 | 10 |
| 180 | COL6A1 | 10 | **366** | PTK7 | 10 |
| 181 | RPL7A | 7 | **367** | FHL1 | 10 |
| 182 | ZNF207 | 9 | **368** | CLEC2B | 10 |
| 183 | TLE3 | 10 | **369** | CCBL2 | 9 |
| 184 | NMB | 9 | **370** | VEGFA | 10 |
| 185 | SRI | 10 | **371** | CLIC1 | 7 |
| 186 | PAICS | 10 | **372** | SEPT9 | 8 |

**Table S2. Significantly under-expressed gene list from Oncomine meta-analysis of normal vs. astrocytoma studies.** *Genes are ranked by significance in Oncomine. **Genes were considered significant in the meta-analysis, and chosen for further analysis, if they were found in at least in at least 7 of 10 meta-analyzed studies.

| Gene Significance Rank* | Gene Symbol | Studies  (of 10)** | Gene Significance Rank* | Gene Symbol | Studies  (of 10)** |
| --- | --- | --- | --- | --- | --- |
|  |  |  |  |  |  |
| 1 | ANK3 | 10 | **139** | RAB11FIP2 | 9 |
| 2 | DCNTN1 | 7 | **140** | DLGAP2 | 9 |
| 3 | BCL2L2 | 10 | **141** | MADD | 10 |
| 4 | WDR7 | 9 | **142** | TNK1 | 9 |
| 5 | PLEKHB2 | 9 | **143** | CD200 | 10 |
| 6 | PRKCZ | 10 | **144** | SORBS2 | 9 |
| 7 | GRM3 | 10 | **145** | RAP1GAP | 10 |
| 8 | OPA1 | 9 | **146** | NR1D1 | 10 |
| 9 | USP12 | 9 | **147** | IDI1 | 10 |
| 10 | ATP6V0A1 | 10 | **148** | MAL | 10 |
| 11 | VAMP1 | 8 | **149** | ELMO1 | 10 |
| 12 | EPB41L3 | 9 | **150** | PURA | 10 |
| 13 | IDH3A | 10 | **151** | KRT17 | 10 |
| 14 | MAPRE2 | 10 | **152** | CD47 | 10 |
| 15 | SH3GL3 | 10 | **153** | RUFY3 | 9 |
| 16 | PTGER3 | 10 | **154** | COX7A1 | 9 |
| 17 | ATP6V1E1 | 9 | **155** | MPHOSPH8 | 9 |
| 18 | RCAN2 | 10 | **156** | BIN1 | 10 |
| 19 | SBF1 | 7 | **157** | C5orf30 | 9 |
| 20 | TSPYL1 | 9 | **158** | CAMTA1 | 9 |
| 21 | RANGAP1 | 9 | **159** | PRDM2 | 10 |
| 22 | ZNF365 | 7 | **160** | GSTM5 | 10 |
| 23 | SNCG | 9 | **161** | EDIL3 | 9 |
| 24 | STAU2 | 9 | **162** | NEFM | 10 |
| 25 | ASPHD1 | 7 | **163** | SYT5 | 10 |
| 26 | PTAFR | 10 | **164** | PRSS3 | 9 |
| 27 | RAB3B | 10 | **165** | LARGE | 9 |
| 28 | PRKCB | 10 | **166** | STK39 | 8 |
| 29 | STMN1 | 10 | **167** | SV2B | 9 |
| 30 | CNNM2 | 9 | **168** | XK | 10 |
| 31 | CACNA1B | 8 | **169** | CLCN4 | 10 |
| 32 | CHIC1 | 7 | **170** | MEG3 | 9 |
| 33 | VAMP2 | 10 | **171** | NECAB3 | 7 |
| 34 | WNT10B | 9 | **172** | YWHAZ | 10 |
| 35 | DUSP7 | 10 | **173** | C22orf9 | 8 |
| 36 | GRIN2C | 10 | **174** | INPP5F | 9 |
| 37 | RUSC2 | 9 | **175** | RAB3A | 10 |
| 38 | MAPK8IP2 | 7 | **176** | DBP | 10 |
| 39 | SSX2IP | 9 | **177** | KCNAB2 | 10 |
| 40 | TPM3 | 9 | **178** | GABRB2 | 8 |
| 41 | TPPP | 9 | **179** | SLC17A7 | 9 |
| 42 | PICK1 | 8 | **180** | AMPH | 10 |
| 43 | APLP1 | 10 | **181** | PDE1A | 10 |
| 44 | GABRA5 | 9 | **182** | QDPR | 10 |
| 45 | SOCS7 | 9 | **183** | FOXO4 | 10 |
| 46 | PPP1R16B | 9 | **184** | KCNMA1 | 10 |
| 47 | TSPAN5 | 9 | **185** | MYH10 | 10 |
| 48 | MAPT | 10 | **186** | CYP26A1 | 9 |
| 49 | MAGI1 | 10 | **187** | RAB40B | 10 |
| 50 | RASGRF1 | 10 | **188** | SEMA4D | 10 |
| 51 | MAST3 | 9 | **189** | NCKIPSD | 9 |
| 52 | CNTN2 | 10 | **190** | PRR4 | 10 |
| 53 | SERPINI1 | 10 | **191** | DCLK1 | 9 |
| 54 | PKP4 | 10 | **192** | UROS | 9 |
| 55 | CACNB1 | 10 | **193** | PDE2A | 9 |
| 56 | NUAK1 | 9 | **194** | KIAA0232 | 10 |
| 57 | SNCA | 10 | **195** | TRIM3 | 8 |
| 58 | KIF17 | 7 | **196** | ARHGEF4 | 8 |
| 59 | GABRA2 | 10 | **197** | SYNPO | 9 |
| 60 | TTBK2 | 7 | **198** | MBOAT7 | 10 |
| 61 | BTRC | 8 | **199** | RIMS2 | 9 |
| 62 | DYNC1I1 | 9 | **200** | TERF2IP | 9 |
| 63 | PRKCG | 10 | **201** | PSD3 | 9 |
| 64 | ZBTB7A | 9 | **202** | STX1A | 10 |
| 65 | LMTK2 | 9 | **203** | ATP6V1C1 | 10 |
| 66 | RYBP | 9 | **204** | FXR2 | 10 |
| 67 | KIF5C | 9 | **205** | RAB40C | 9 |
| 68 | AGTPBP1 | 9 | **206** | KIT | 10 |
| 69 | RYR2 | 10 | **207** | UBE3A | 10 |
| 70 | LDOC1 | 8 | **208** | ZC3H13 | 9 |
| 71 | TPD52 | 10 | **209** | CLASP2 | 9 |
| 72 | MOBP | 10 | **210** | MAN1A2 | 9 |
| 73 | TUBB4 | 10 | **211** | HLF | 10 |
| 74 | LDB3 | 8 | **212** | SYN2 | 10 |
| 75 | CACNA1A | 9 | **213** | NEBL | 9 |
| 76 | BSCL2 | 9 | **214** | NR2F6 | 9 |
| 77 | MPP2 | 9 | **215** | CPLX2 | 10 |
| 78 | PPP2R2B | 10 | **216** | JAKMIP1 | 8 |
| 79 | RAP1GDS1 | 9 | **217** | MYCBP2 | 9 |
| 80 | GOT1 | 10 | **218** | DGKZ | 10 |
| 81 | WASF1 | 10 | **219** | DOCK3 | 9 |
| 82 | FAIM2 | 9 | **220** | KCNC3 | 7 |
| 83 | FCH01 | 9 | **221** | HABP4 | 9 |
| 84 | CDKN2D | 10 | **222** | PRKCQ | 9 |
| 85 | EFR3B | 9 | **223** | SLC13A3 | 9 |
| 86 | MBP | 10 | **224** | RAPGEF5 | 8 |
| 87 | SNRPN | 10 | **225** | PET112L | 8 |
| 88 | CA11 | 9 | **226** | KCNC4 | 10 |
| 89 | SERINC3 | 10 | **227** | GRM7 | 8 |
| 90 | MICAL3 | 9 | **228** | NPY1R | 10 |
| 91 | EFNA5 | 9 | **229** | CCKBR | 10 |
| 92 | FGF13 | 9 | **230** | DLG1 | 10 |
| 93 | IQSEC1 | 9 | **231** | GRM1 | 8 |
| 94 | PAK1 | 10 | **232** | PEBP1 | 10 |
| 95 | ULK2 | 9 | **233** | RNMT | 9 |
| 96 | ATP2B1 | 10 | **234** | CCDC64 | 8 |
| 97 | MAP2K4 | 10 | **235** | PEG3 | 10 |
| 98 | GABARAPL1 | 9 | **236** | GRLF1 | 10 |
| 99 | APBB1 | 10 | **237** | KLK3 | 10 |
| 100 | ATP8A1 | 9 | **238** | NAV3 | 8 |
| 101 | AUH | 10 | **239** | CLDN9 | 9 |
| 102 | NIPAL3 | 10 | **240** | PRKAR1A | 10 |
| 103 | DNM1L | 10 | **241** | CRHR2 | 8 |
| 104 | EMX1 | 9 | **242** | PPP1R7 | 10 |
| 105 | SCAMP1 | 10 | **243** | ICAM5 | 10 |
| 106 | FAAH | 10 | **244** | CRELD1 | 8 |
| 107 | LPGAT1 | 10 | **245** | PRKCE | 10 |
| 108 | PTPRD | 10 | **246** | ARFGEF2 | 8 |
| 109 | GHITM | 8 | **247** | PPFIA2 | 9 |
| 110 | AAK1 | 9 | **248** | FABP6 | 9 |
| 111 | DGK1 | 10 | **249** | PDIA2 | 9 |
| 112 | CACNA1C | 10 | **250** | OSBPL1A | 9 |
| 113 | FUT9 | 9 | **251** | RAPGEF3 | 10 |
| 114 | GRIN2A | 10 | **252** | FAM190B | 10 |
| 115 | PDS5B | 8 | **253** | CALM1 | 9 |
| 116 | KCNJ9 | 8 | **254** | LOC157627 | 8 |
| 117 | APBA1 | 10 | **255** | PCDH9 | 9 |
| 118 | DIP2C | 9 | **256** | DOCK9 | 9 |
| 119 | IQSEC3 | 9 | **257** | FRMPD4 | 8 |
| 120 | TYRO3 | 9 | **258** | HSPH1 | 10 |
| 121 | KIAA0284 | 7 | **259** | C17orf108 | 9 |
| 122 | HK1 | 7 | **260** | SEPT11 | 9 |
| 123 | PAFAH1B1 | 10 | **261** | DNAJC6 | 9 |
| 124 | FXYD1 | 9 | **262** | GDE1 | 9 |
| 125 | SLC25A4 | 10 | **263** | EXOC6B | 7 |
| 126 | KHDRBS2 | 7 | **264** | CAMK2G | 10 |
| 127 | HRAS | 10 | **265** | OPTN | 9 |
| 128 | ACTR1A | 10 | **266** | ANXA3 | 9 |
| 129 | AP3M2 | 10 | **267** | PPFIA3 | 9 |
| 130 | MEF2C | 10 | **268** | NDEL1 | 9 |
| 131 | CLTB | 9 | **269** | CDC42 | 9 |
| 132 | ATP2B3 | 10 | **270** | CDH8 | 10 |
| 133 | TLN2 | 10 | **271** | ARPP19 | 9 |
| 134 | SEC14L5 | 9 | **272** | DTNB | 9 |
| 135 | SLC6A12 | 10 | **273** | PPM1A | 10 |
| 136 | RB1CC1 | 10 | **274** | ATP58 | 10 |
| 137 | ATP5L | 7 | **275** | MTMR9 | 9 |
| 138 | SPOCK3 | 7 |  |  |  |

**Table S3. Significant over-represented GO Terms for differentially expressed genes in Astrocytoma.**

| Rank | p-adjusted | GO ID | GO Name |
| --- | --- | --- | --- |
| 1 | 0.006 | GO:0019829 | Cation-transporting ATPase activity |
| 2 | 0.036 | GO:0033267 | Axon part |
| 3 | 0.04 | GO:0005938 | Cell cortex |
| 4 | 0.04 | GO:0014069 | Postsynaptic density |
| 5 | 0.013 | GO:0044420 | Extracellular matrix part |
| 6 | 0.019 | GO:0032990 | Cell part morphogenesis |
| 7 | 0 | GO:0032989 | Cellular component morphogenesis |
| 8 | 0 | GO:0019900 | Kinase binding |
| 9 | 0 | GO:0044456 | Synapse part |
| 10 | 0.011 | GO:0019901 | Protein kinase binding |
| 11 | 0.001 | GO:0010035 | Response to inorganic substance |
| 12 | 0.002 | GO:0007268 | Synaptic transmission |
| 13 | 0 | GO:0043005 | Neuron projection |
| 14 | 0 | GO:0044419 | Interspecies interaction between organisms |
| 15 | 0.002 | GO:0044057 | Regulation of system process |
| 16 | 0.013 | GO:0022603 | Regulation of anatomical structure morphogenesis |
| 17 | 0 | GO:0019899 | Enzyme binding |
| 18 | 0 | GO:0008092 | Cytoskeletal protein binding |
| 19 | 0 | GO:0007155 | Cell adhesion |
| 20 | 0 | GO:0022610 | Biological adhesion |
| 21 | 0 | GO:0042127 | Regulation of cell proliferation |
| 22 | 0 | GO:0042995 | Cell projection |
| 23 | 0 | GO:0005515 | Protein binding |
| 24 | 0.007 | GO:0005509 | Calcium binding |
| 25 | 0 | GO:0008150 | Biological process |
| 26 | 0.001 | GO:0010033 | Response to organic substance |
| 27 | 0 | GO:0023034 | Intracellular signaling pathway |
| 28 | 0 | GO:0044459 | Plasmamembrane part |
| 29 | 0.013 | GO:0045907 | Intracellular transport |
| 30 | 0.001 | GO:0009653 | Anatomical structure morphogenesis |
| 31 | 0.011 | GO:0050793 | Regulation of developmental process |
| 32 | 0.011 | GO:0051239 | Regulation of multicellular organismal process |
| 33 | 0.016 | GO:0010646 | Regulation of cell communication |
| 34 | 0.013 | GO:0035466 | Regulation of signaling pathway |
| 35 | 0.035 | GO:0045184 | Establishment of protein localization |
| 36 | 0.035 | GO:0042981 | Regulation of apoptosis |
| 37 | 0 | GO:0005488 | Binding |
| 38 | 0.033 | GO:0050790 | Regulation of catalytic activity |
| 39 | 0.015 | GO:0065009 | Regulation of molecular function |
| 40 | 0.03 | GO:0031226 | Intrinsic to plasma membrane |
| 41 | 0 | GO:0048518 | Positive regulation of biological process |
| 42 | 0.006 | GO:0042221 | Response to chemical stimulus |
| 43 | 0 | GO:0048522 | Positive regulation of cellular process |
| 44 | 0.015 | GO:0065008 | Regulation of biological quality |
| 45 | 0 | GO:0043234 | Protein complex |
| 46 | 0.001 | GO:0048519 | Negative regulation of biological process |
| 47 | 0 | GO:0009987 | Cellular process |
| 48 | 0.016 | GO:0048856 | Anatomical stucture development |
| 49 | 0.011 | GO:0048523 | Negative regulation of cellular process |
| 50 | 0 | GO:0005737 | Cytoplasm |
| 51 | 0 | GO:0032502 | Developmental process |
| 52 | 0.002 | GO:0005886 | Plasma membrane |
| 53 | 0.007 | GO:0016043 | Cellular component organization |
| 54 | 0.005 | GO:0023052 | Signaling |
| 55 | 0 | GO:0044444 | Cytoplasmic part |
| 56 | 0.006 | GO:0032991 | Mactromolecular complex |
| 57 | 0 | GO:0065007 | Biological regulation |
| 58 | 0.002 | GO:0050789 | Regulation of biological process |
| 59 | 0.005 | GO:0050794 | Regulation of cellular process |
| 60 | 0.013 | GO:0044424 | Intracellular part |
